# Guide to lower extremity radiologic measurements: part 1 hip

**DOI:** 10.1007/s00256-026-05189-0

**Published:** 2026-04-06

**Authors:** Allison M. Bronson, Imran M. Omar, Allison M. Crone, Ryan S. Selley, Jennifer S. Weaver, Andrea S. Klauser, Mihra S. Taljanovic

**Affiliations:** 1 Department of Radiology, Feinberg School of Medicine, Northwestern University, 676 North Saint Clair St., Suite 800, Chicago, IL 60611 USA; 2 Department of Radiology and Imaging Sciences, Indiana University School of Medicine, 714 North Senate Ave., Suite 200, Indianapolis, IN 46202 USA; 3 Department of Orthopaedic Surgery, Feinberg School of Medicine, Northwestern University, 676 North Saint Clair St., Suite 1350, Chicago, IL 60611 USA; 4 Department of Radiology, 7703 Floyd Curl Drive, University of Texas at San Antonio, San Antonio, TX 78229 USA; 5 Department of Radiology, Innsbruck Medical University, Privatpraxis Bruneckerstr 2E/5. Stock, Innsbruck, Austria; 6 Departments of Radiology and Imaging Sciences and Orthopaedic Surgery, College of Medicine, The University of Arizona, 1501 North Campbell Ave, Tucson, AZ 85724 USA; 7 Department of Radiology, MSC 10 5530, 1 University of New Mexico, Albuquerque, NM 87131 USA

**Keywords:** Hip Joint, Measurement, Radiography, Computed tomography, Magnetic resonance imaging, Dysplasia

## Abstract

Potential etiologies of hip pain, especially in younger patients with acetabular dysplasia and various impingement syndromes, or in older patients following arthroplasty can be subtle radiographically, and quantitative assessment on standardized imaging is often performed to confirm the diagnosis and help clinicians choose between conservative or surgical management. As hip preservation and joint replacement surgical techniques have become more sophisticated and widespread, the number of measurements reported in the literature to detect subtle abnormalities has greatly increased, and measurement techniques may vary between institutions. As a result, musculoskeletal radiologists may be less familiar with particular measurements requested by clinicians. This article is the first of a three-part series describing measurements of the lower extremity and focuses on the most common measurements used by hip preservationists and hip replacement surgeons, including the proper study on which to perform each measurement, the proper measurement technique and the normal value(s) based on current literature. Finally, the implications of abnormal values for each measurement are briefly discussed. The measurements are grouped by anatomy and pathology; those that are more commonly used clinically and reported in the literature are discussed in greater depth, while those that are less common or may be primarily used for research are described more briefly with imaging examples.

## Introduction

Although qualitative assessment of diagnostic imaging is often sufficient to establish many musculoskeletal diagnoses in the hip and lower limb, there are situations in which quantitative measurements can be helpful, for example in detecting subtle abnormalities of acetabular dysplasia or providing supportive evidence of femoroacetabular impingement. In these instances, measurements can help establish the degree of abnormality, which can guide treatment decisions, including the necessity of surgical intervention and the appropriate degree of correction. In cases of failing hip prostheses, detecting deviations in initial positioning of the device components can provide insight into why particular devices may have started to fail.

For any measurement, it is important to understand the proper measurement technique, its normal values, and the implications of abnormal values. Moreover, it is critical to utilize measurements with well-established normal values and measurement protocols to standardize quantitative assessment and avoid inaccurate measurements that may lead to errors in management. This review article will discuss many of the commonly utilized measurements of the lower limb as a whole and measurements specific to the hip, as well as the appropriate imaging studies on which to assess them.

## Lower limb

### Imaging modalities

#### Radiography

Full-limb imaging is the primary tool for evaluating leg length discrepancy and measuring leg alignment to define the physiologic, anatomic, and mechanical axes and angles of the lower limb joints. The patient should be positioned standing, with the patellae centered over the femoral condyles [1]. Historically, the standing AP full-limb radiograph, also known as a teleoroentgenogram, was the most common method of imaging the lower limbs and consisted of a single direct radiographic exposure of the entire lower limb with the x-ray beam centered at the knee. The lower extremities were in a neutral position with the patellae facing the x-ray beam. This technique, however, results in significant magnification error due to beam divergence. To minimize magnification error, the “scanogram” technique was introduced consisting of three separate exposures centered over the hip, knee, and ankle joints with the patient next to a calibrated ruler.

In the age of computed radiography-based radiographic imaging, there are two common methods of image acquisition [2]. The first is a modified standing AP full-limb radiograph, which uses three radiographic exposures that are digitally stitched together to form one image. The second is a three-dimensional (3D) biplanar radiograph imaging system (EOS®), which scans the body with two perpendicular thin x-ray beams resulting in simultaneous acquisition of the anteroposterior (AP) and lateral views and allows for the possibility of 3D reconstruction. While both techniques accurately measure limb angles, the modified standing AP full-limb radiograph results in magnification error due to beam divergence [2], so these images should be performed with a radiopaque ruler for measurement calibration. On the other hand, the 3D biplane radiograph avoids this distortion.

#### Cross-sectional imaging

For patients with torsional malalignment of the lower limb, axial cross-sectional evaluation is the gold standard for preoperative assessment. The patient is positioned supine with the legs fully extended. The long axis of the limb should be positioned parallel to the long axis of the scanner to produce accurate measurements, though with newer reconstructive techniques this can often be corrected in postprocessing. If the limb cannot be straightened due to contracture or osseous deformity, reconstructed true axial images of each area of interest should be used for measurement.

Either computed tomography (CT) or magnetic resonance imaging (MRI) can be used when needed. However, CT has several advantages over MRI when assessing measurements, such as lower extremity torsion. CT has exquisite bone trabecular detail, a generally lower cost, and is more widely available. Although it does involve exposing patients to ionizing radiation, modern scanners and low-dose protocols have resulted in significant dose reduction, making doses negligible. Thin section axial CT is often performed in three discrete segments through the hip, knee, and ankle, rather than scanning the entire lower limb, which can also reduce the overall radiation dose and the number of acquired images. Perhaps most importantly, CT is much faster than MRI, which can greatly reduce the risk of motion when imaging the different segments and improve the accuracy of rotational measurements, such as version angles. Finally, CT imaging protocols may be easier to standardize between institutions, allowing interinstitutional comparison.

MRI can also be used to evaluate torsion [3]; however, care must be taken to ensure that the patient does not move during the longer scan time to ensure accurate measurement. Compared with CT, MRI provides much better contrast resolution, which can help better detect marrow abnormalities, such as trabecular fractures or marrow replacement, and soft tissue pathologies, such as fluid collections or ligamentous injuries.

### Measurements

#### Leg length

Inequality in leg length can result in compensatory gait abnormalities and accelerated degenerative lower extremity arthritis and spondylosis. Leg length discrepancy can be categorized as anatomic or functional. Anatomic leg length discrepancy (LLD) is when the cumulative bone length and cartilage thickness significantly differ between legs such as in hemihypertrophy or fracture deformities. Functional leg length discrepancy can be related to angular and torsional deformities, as well as soft tissue contractures. For example, flexion contractures about the hip and knee can result in apparent shortening of the leg, while abduction contractures of the hip and equinus deformity of the ankle can functionally lengthen the leg [4].

The functional leg length is measured on full-limb radiographs as the distance between the superior margin of the femoral head to the center of the tibial plafond [5], though many alternative reference points, such as the anterior superior iliac spine, the femoral head center, and the talar dome, have been described. It has been reported that approximately 75% of adults have a physiologic lower limb length discrepancy of ≤ 7 mm [6].

The anatomic femoral length can be described as the distance between the superior margin of the femoral head to the center of the medial femoral condyle articular margin. The anatomic tibial length can be described as the distance between the lowest point of the intercondylar eminence and the center of the tibial plafond. The anatomic leg length is the sum of the anatomic femoral and tibial lengths [4].

While full-length AP standing radiographs are the gold standard of total limb length measurement, the degree of limb length discrepancy related to the hip can be measured on AP standing pelvis radiographs, also known as the “regional limb length” which is described later in the hip arthroplasty section.

#### Alignment

Proper alignment of the lower extremities is crucial to evenly distributing forces throughout the knee (Table 1). Asymmetric mechanical load can result in locally greater forces and lead to asymmetric cartilage loss and osteoarthritis (OA). Torsional abnormalities impact the biomechanics of gait, clinically detected by alterations in the foot progression angle [7]. Additionally, it is important to correct any imbalances during joint replacement to prevent hardware failure. As with assessment of limb length, angular measurements of alignment are routinely performed with standing, weightbearing radiographs of the lower extremities or newer modalities, such as EOS®.

**Table 1:** Commonly used measurements in the radiologic assessment of the lower extremity

Radiologic measurement	Definition	Imaging modality of choice	Normal value(s)/normal range	Abnormal value(s)/clinical significance
Leg length discrepancy	Anatomic leg length is the vertical distance between the apex of the femoral head and the central tibial plafond. Discrepancy is the difference between right and left sides	Full-length AP standing radiographs or 3D-biplanar radiograph	< 10 mm generally well tolerated	> 10 mm can lead to gait disturbances, muscle imbalance and accelerated joint degeneration
Hip-knee-ankle angle	Angle formed between the femoral and tibial mechanical axes (Fig. 1)	Full-length AP standing radiographs or 3D-biplanar radiograph	1º–1.5° varus angulation	**·** > 1º–1.5° varus angulation can lead to excessive medial compartment load bearing **·** < 1º–1.5° varus angulation or valgus angulation can lead to excessive lateral compartment load bearing
Femoral-shaft tibial-shaft angle	Angle formed between the femoral and tibial anatomic axes (Fig. 1)	Full-length AP standing radiographs or 3D-biplanar radiograph	173º–175° if measured obtusely 5º–7° if measured acutely	**·** > 175° (obtuse) or < 5° (acute) genu varum **·** < 173° (obtuse) of > 7° (acute) genu valgum
Femoral torsion angle	Angle formed between lines along the femoral neck long axis and posterior femoral condyles (Fig. 2)	Axial CT or MRI through hip and knee	•4º–17° normal • < 8° difference in sides	**·** > 17° excessive femoral antetorsion **·** < 4° decreased femoral antetorsion or even retrotorsion
Tibial torsion angle	Angle formed by a line connecting posterior medial and lateral tibial condyles at the physeal level and a line bisecting the medial and lateral malleoli (or the tibial plateau at the incisura fibularis) (Fig. 3)	Axial CT or MRI through hip and knee	19º–25°	**·** > 25° excessive tibial external torsion **·** < 19° excessive tibial internal torsion

##### Anatomic axis

**Fig. 1:**
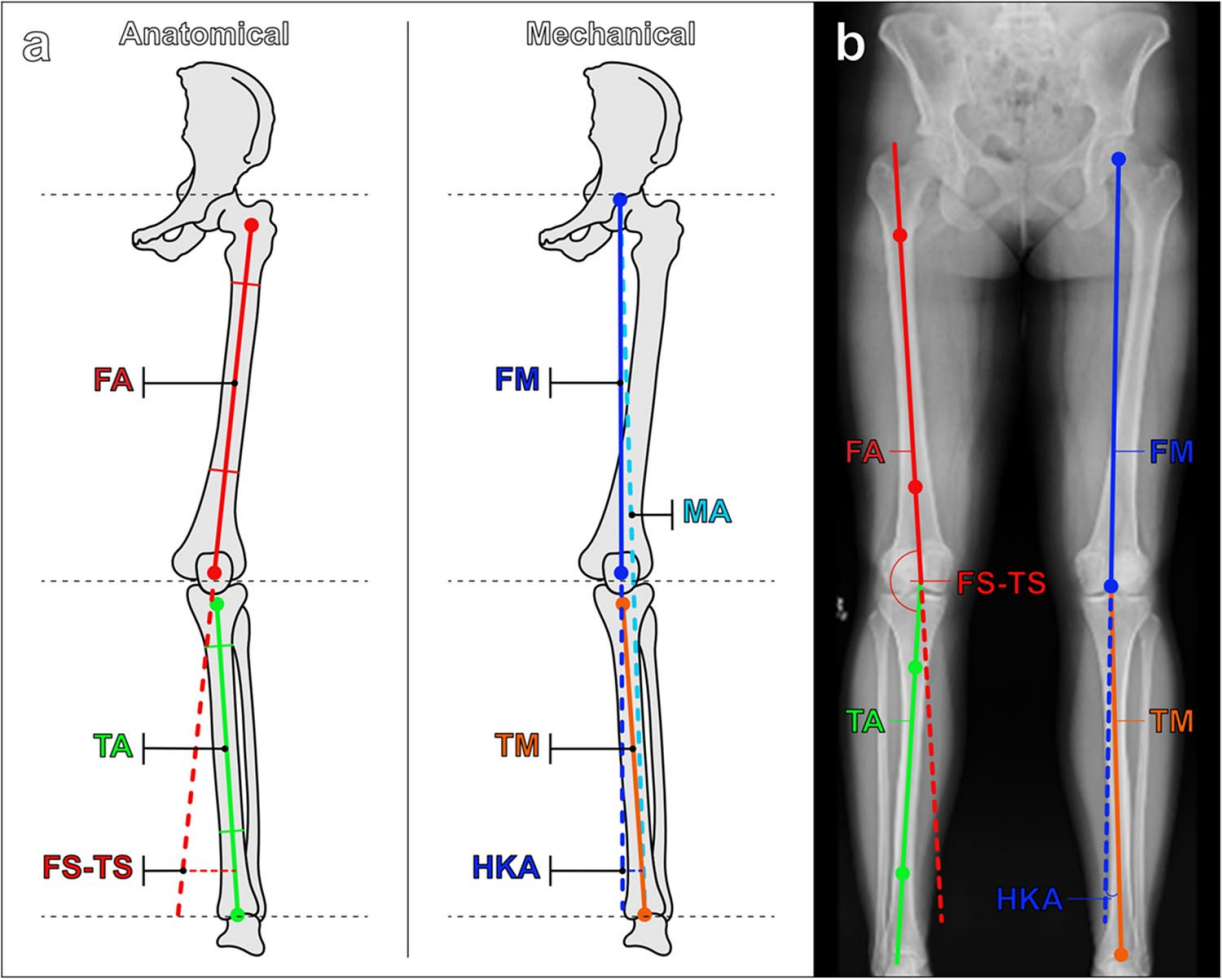
Measurement of lower extremity axes schematically and on weightbearing full-limb radiographs. **a** Diagrams showing the major axes of the lower limb. The anatomic axis of a bone is measured by drawing a line between two central intramedullary points within the proximal and distal diaphysis of the bone, for example the femoral axis (FA, red line) and tibial axis (TA, green line). The mechanical axis (FM, dark blue line) of the femur extends from the center of the femoral head to the central intercondylar notch, while the mechanical axis of the tibia (TM, orange line) is measured from the center of the tibial interspinous groove to the central tibial plafond. Finally, the mechanical axis of the lower limb (MA, dashed light blue line) is determined by creating a line connecting the center of the femoral head and the central tibial plafond. The femoral-shaft tibial shaft angle (FS-TS), also known as the anatomic tibiofemoral angle, represents the obtuse angle between the anatomic axes of the femur (FA) and the tibia (TA). The hip-knee-ankle angle (HKA), or the mechanical tibiofemoral angle, is the acute angle formed by the femoral and tibial mechanical axes and is important in assessing coronal alignment for varus or valgus angulation, which can result in accelerated osteoarthritis or abnormal stress on knee arthroplasties. **b** Whole-limb standing AP EOS® image depicts the various angles used to assess lower limb alignment

The anatomic axis of a bone represents the long axis of the diaphysis of a long bone. It should be measured on large field-of-view survey imaging of the lower extremities. The axis is measured by drawing a line between two intramedullary points within the proximal and distal diaphysis of the bone (Fig. 1). The points should be midway between the medial and lateral outer cortical surfaces of the bone and about 10 cm from the nearest joint. The anatomic femoral and tibial axes are not parallel due to the natural angulation of the femur. Clinically, the anatomic axis is used to guide surgical procedures, such as intramedullary nailing, limb length correction, and osteotomies to correct complex deformities.

##### Mechanical axis

The mechanical axis of a bone represents the load-bearing axis. The mechanical axis of the femur extends from the center of the femoral head to the central intercondylar notch, while the mechanical axis of the tibia is measured from the center of the tibial interspinous groove to the central tibial plafond (Fig. 1). The anatomic axis of the femur is in approximately 6° ± 1° of valgus angulation compared to the mechanical axis, known as the anatomic-mechanical femoral angle [1]. On the other hand, the mechanical axis of the tibia is normally parallel to the anatomic axis of the tibia but located slightly lateral, as the anatomic axis typically intersects the knee joint line at the medial tibial spine. The difference between the anatomic and mechanical axes may allow more even load distribution with weightbearing in the knee and may help to smooth movement in the knee during flexion and rotation, resulting in smoother gait.

The mechanical axis of the lower limb is drawn from the center of the femoral head to the center of the tibial plafond. This runs approximately 3° oblique, from craniolateral to mediocaudal, to the axis of the body. The line’s physiologic position runs on average 2–6 mm medial to the center of the knee. The distance from the mechanical axis to the center of the knee joint is called the mechanical axis deviation (MAD). When the mechanical axis is > 15 mm medial to the center of the knee joint, this represents clinically significant varus angulation, while > 10 mm lateral deviation represents valgus angulation [1]. The mechanical axis is most commonly used to plan total knee arthroplasty alignment, to ensure appropriate forces on the device during weightbearing and locomotion. However, some newer surgical techniques may use the anatomic axis to tailor prostheses to particular patients. 

##### Hip-knee-ankle angle

The hip-knee-ankle angle, or the mechanical tibiofemoral angle, is the acute angle formed by the femoral and tibial mechanical axes as measured on full-length lower limb radiographs (Fig. 1). Normal measurements are approximately 1º–1.5° of varus angulation [8]. The hip-knee-ankle angle is used to assess knee alignment, most commonly in patients with knee OA to characterize the degree and progression of deformity and guide surgical correction, or following knee arthroplasty.

##### Femoral-shaft/tibial-shaft angle

The femoral-shaft/tibial-shaft angle, also known as the anatomic tibiofemoral angle, is usually reported as the obtuse angle between anatomic axes of femur and tibia as measured on full-length lower limb radiographs (Fig. 1), although occasionally, it can be reported as the acute, supplementary angle [1]. While weightbearing AP knee radiographs are sometimes used clinically to estimate the femorotibial angle, this has been shown to be inaccurate when compared to measurements made on full limb radiographs, especially if the knee is not fully extended [9]. In adults, this angle measures between 173° and 175° if measured obtusely, or 5º–7° if the acute angle is used. Genu valgum is diagnosed when the measured angle is < 173° when the obtuse angle is used and > 7° if measured acutely, while genu varum is diagnosed when the angle is > 175° when the obtuse angle is used and < 5° if measured acutely. Genu varum is physiologic at birth and reaches its peak between 6 and 12 months of age. During normal growth the femoral-shaft/tibial-shaft angle decreases, reaching 180° using the obtuse angle (or 0° when using the acute angle) between 18 and 24 months. At age 2 years, this becomes a physiologic genu valgum reaching the adult configuration by 6 to 7 years of age [10]. Genu varum after the age of 2 years is considered to be abnormal. The hip-knee-ankle angle (mechanical axis) is generally about 5° different than the femoral-shaft tibial-shaft angle (anatomic axis) when the acute angle measurement is used [11].

##### Femoral torsion angle

Torsion is technically a measurement of the rotation of a long bone along its longitudinal axis, while version typically refers to the orientation of a joint along its transverse axis. Most commonly, the version angle refers to the actual measurement, while torsion refers to the state of having an abnormal measurement. However, the terms femoral version and femoral torsion are often used interchangeably in the literature. In 2020, the Lisbon Agreement on Femoroacetabular Impingement concluded that MRI is the gold standard modality for comprehensive assessment of hip impingement and should include measurement of femoral torsion. Although this group did not provide specific parameters, they advised fast axial imaging through the hip and knee through the femoral condyles should be routinely performed [12].

**Fig. 2:**
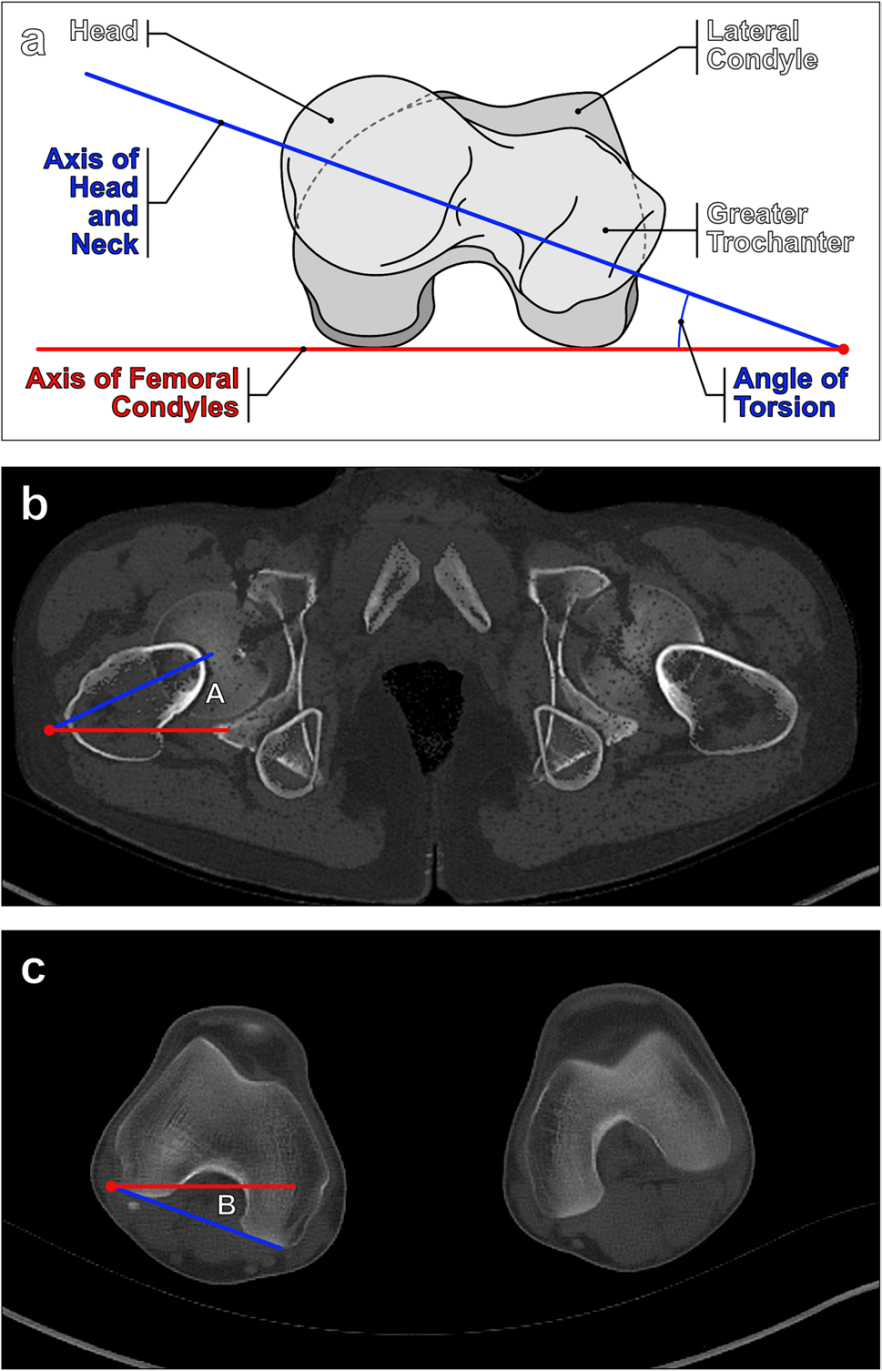
Measurement of femoral torsion angle on axial CT of the lower extremity. **a** Diagrammatic representation of the femur from a top-down perspective, which illustrates the concept of femoral torsion. One line is drawn through the femoral neck axis (blue line), while the other drawn along the posterior femoral condyles (red line). **b** On axial CT of the pelvis with the greater trochanters superimposed over the femoral heads, an angle (*A*) is formed between a line along the axis of the femoral neck (blue line) and a horizontal line (red line). **c** On axial CT at the level of the distal femoral condyles, an angle (*B*) is formed between a line along the posterior femoral condyles (blue line) and a horizontal line (red line). The femoral torsion angle is calculated by combining *A* and *B* relative to the horizontal. If the femoral condyles are internally rotated with respect to a horizontal reference line, the resulting angle is added to the femoral neck long axis angle. If the femoral condyles are externally rotated with respect to a horizontal reference line, the resulting angle is subtracted from the femoral neck long axis angle. Normal adult femoral antetorsion is approximately 4–17° and differences between sides should be < 8°

The femoral torsion angle is formed most commonly on axial imaging of the proximal and distal femur by two lines: a line along the femoral neck long axis and a line connecting the posterior margins of the femoral condyles (Fig. 2) [13]. If performed on postprocessed axial images with the femoral head/neck superimposed on the femoral condyles, the angular measurement is straightforward. If performed on two separate axial images, the lines are drawn with respect to a reference horizontal line, and the torsion is calculated from these angles. This is accomplished by first measuring the angle formed by the femoral neck long axis relative to horizontal. Then, if the femoral condyles are internally rotated with respect to a horizontal reference line, the resulting angle is added to the femoral neck long axis angle. If the femoral condyles are externally rotated with respect to a horizontal reference line, the resulting angle is subtracted from the femoral neck long axis angle.

Several methods have been described, differing slightly with regard to anatomical landmarks and the positioning of imaging axes with different resulting normal values. The anatomic femoral neck axis can be difficult to accurately define, as the lateral part of the femoral neck is elliptical and anteriorly tilted, and the femoral head is not usually centered on the femoral shaft [14]. Therefore, drawing the femoral neck axis on axial images has been shown to underestimate the degree of femoral torsion. The measurement is more accurately performed on reformatted oblique images through the femoral head-neck. Measurements on CT, MRI, and 3D biplanar radiography reconstructions have been shown to be equivalent [13].

At birth, the femoral torsion is 34º–40°, which decreases during normal growth [15]. Using the oblique slice orientation method of measurement, normal adult femoral antetorsion is approximately 4º–17° and differences between sides should be < 8° [16]. Abnormal femoral torsion angles are associated with altered biomechanics, resulting in abnormal force distribution, particularly in the hip. Excessive femoral antetorsion can cause posterior extraarticular impingement, which can result in compensatory lower limb internal rotation, or in-toeing. Heimann et al. hypothesized that excessive femoral antetorsion places increased stress on the anterior hip and can result in greater incidence of anterior labral tearing, while excessive femoral retrotorsion puts additional stress on the posterior hip and may be more frequently associated with posterior labral injury [14, 17].

Femoral torsion also has implications for biomechanics at the knee. For example, a recent study by Guggenberger et al. found that increased femoral antetorsion can increase lateral patellofemoral loading [18], while Barton et al. concluded that femoral internal torsion >25° should be considered for axial alignment correction in patellofemoral instability [19].

##### Tibial torsion angle

The tibial torsion angle represents the rotation of the tibia along its long axis; an increased torsion angle is called external tibial torsion and causes the foot to rotate outward (out-toeing), while a decreased torsion angle is called internal tibial torsion and causes the foot to rotate inward (in-toeing). Both can impact gait. Furthermore, tibial torsion abnormalities can impact mediolateral knee kinematics at the patellofemoral articulation, with recent evidence suggesting higher tibial torsion angles are associated with greater lateral patellofemoral forces [18].

**Fig. 3:**
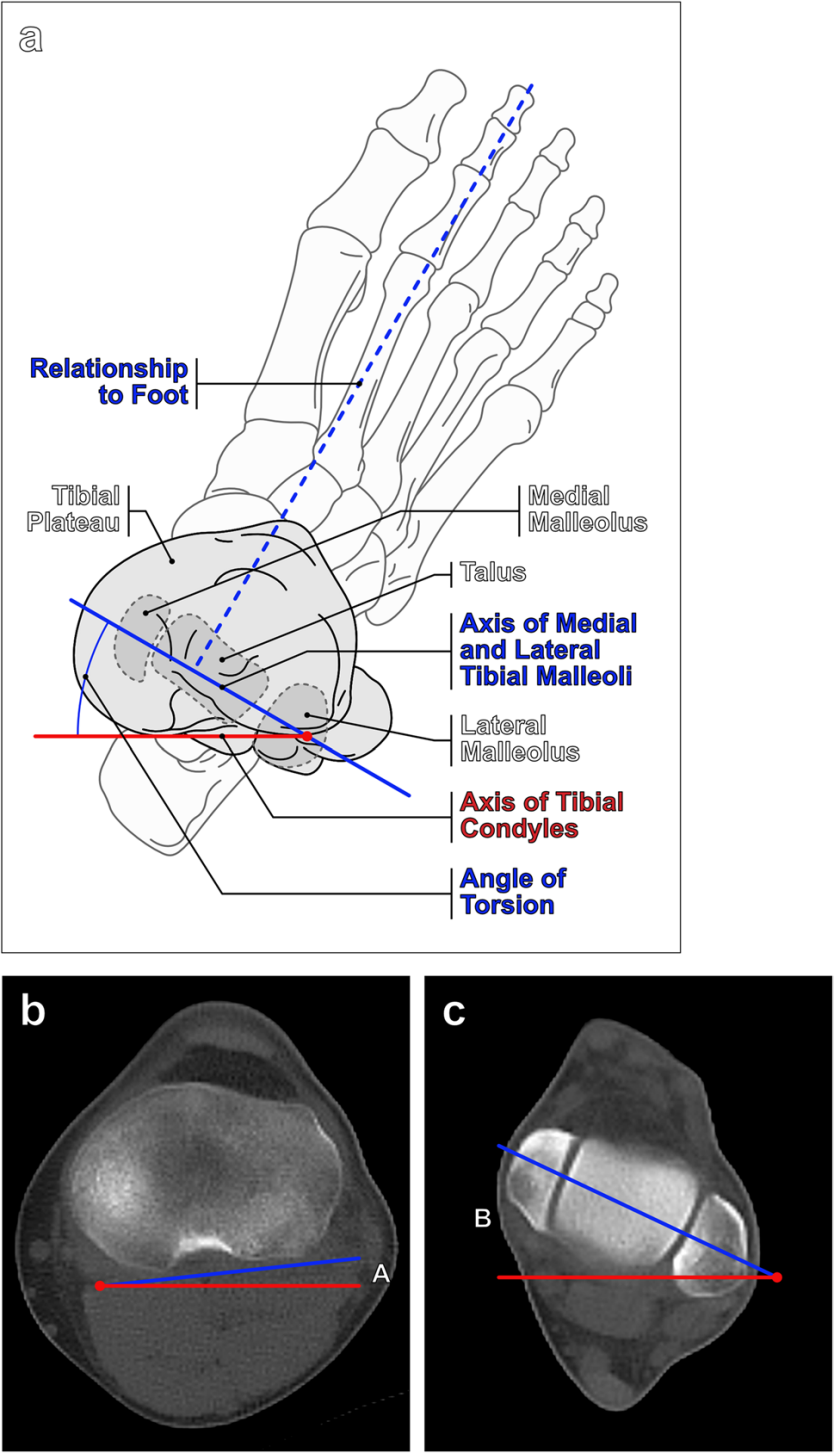
Measurement of tibial torsion angle schematically and on axial CT imaging. **a** Diagram illustrating the determination of the tibial torsion angle. The axis of the tibial condyles (red line) is drawn along the posterior margin of the medial and lateral tibial condyles, while the distal tibial axis is drawn by bisecting the medial and lateral malleoli (blue line). **b** Axial CT image of the proximal left tibia shows the angle (*A*) formed between a line along the posterior margin of the tibial condyles (blue line) and a horizontal line (red line). **c** On axial CT image at the level of the ankle, angle (*B*) is formed between a line bisecting the medial and lateral malleoli (blue line) and the horizontal (red line). The tibial torsion angle is calculated by combining angles *A* and *B* relative to the horizontal; internal rotation is conventionally reported with negative values, and the upper (proximal tibial) angle is usually slightly internally rotated. On the other hand, external rotation is generally reported with positive values, and the lower (intermalleolar angle) is usually externally rotated. The upper angle is subtracted from the lower angle. A tibial torsion of 19º–25° is generally considered normal, while an angle > 30° reflects excessive external tibial torsion, and differences of > 15° from side to side are abnormal

Multiple methods of measurement have been reported in the literature with different anatomic landmarks along the distal femur, tibia, and talus. The most commonly reported methods of measuring the proximal tibial axis are (1) a line bisecting the tibial plateau through its center or (2) a line connecting the posterior condyles of the tibial plateau. Both are best measured at the level of the physeal line and demonstrate equivalent reliability [20]. The most common method of measurement for the distal tibial axis is a line bisecting the medial and lateral malleoli [21]. A small percentage of authors describe the distal tibial axis as a line bisecting the medial malleolus extending through the incisura fibularis, though with lower inter- and intraobserver reliability [22]. The tibial torsion angle is then calculated by combining the proximal and distal tibial axes relative to the horizontal line (Fig. 3); internal rotation is conventionally reported as having a negative measurement, while external rotation is generally reported with positive values. Generally, the proximal tibia is slightly internally rotated relative to the horizontal axis while the distal tibia is externally rotated, so the tibial torsion angle is calculated by subtracting the absolute value of the upper, proximal tibial angle from the lower, intermalleolar angle. If the proximal tibia is externally rotated, the angle would be calculated by adding the two positive numbers.

Clinically, tibial torsion is measured in younger patients with gait abnormalities to determine whether conservative measures, such as bracing, are appropriate or surgical techniques, such as derotational osteotomies are required. In patients with knee OA, calculation of both femoral and tibial torsion angles is often used preoperatively to customize device positioning for patients. Similarly, these values can be assessed following knee arthroplasty to determine whether rotational alignment could contribute to implant failure.

At birth, tibial torsion is approximately 2°, rapidly increasing in early childhood when it reaches adult values in the first 1–4 years of life [23]. While there is high variability between articles in defining mean values based on differing measurement techniques, multiple studies using the transmalleolar technique report an average torsion of 19º–25° [21]. Differences of > 15° from side to side are abnormal [24]. The most commonly reported surgical threshold is > 30° of external tibial torsion [21].

## Hip

### Imaging modalities

#### Radiography

Radiography is a mainstay of qualitative assessment of the hip and requires highly standardized positioning to allow for accurate measurements. The most important projection is the orthograde AP pelvic radiograph. Because weightbearing offers a more physiologic assessment of the hip than supine positioning, this radiograph should be obtained with the patient standing, if possible, with both lower extremities in 15º–20° of internal rotation to compensate for normal femoral antetorsion. When going from a supine to a standing position, the pelvis tends to tilt posteriorly, which can increase the measured acetabular version angle [25]. The view is deemed technically adequate when the tip of the coccyx is centered and located 1–3 cm above the superior margin of the pubic symphysis. Adherence to these parameters prevents excessive pelvic rotation and abnormal tilting, which can distort the anatomy and lead to measurement errors.

The false profile view is particularly useful in assessing anterior femoral head coverage by the superior, weightbearing region of the joint, and measuring the anterior center-edge angle. This view is obtained with the patient standing with the affected hip against the detector and angled at 65°. The foot ipsilateral to the side of the affected hip is laterally rotated so that it is parallel to the image detector. The view is deemed technically adequate when the distance between the two femoral heads is approximately the size of one femoral head. An example of the false profile view is seen in Fig. 11, which discusses the anterior center-edge angle.

Other projections are helpful adjunctive views. For example, although they are not as commonly used in quantitative assessment, the Dunn, frog-leg lateral, and cross-table lateral radiographs are used to measure the alpha angle in order to assess femoral head sphericity and the anterior aspect of the femoral head-neck junction for cam morphology [26, 27]. Several recent works have shown very good concordance between deep learning-based automated measurement applications and expert manual measurements when using AP and false profile pelvic radiographs to generate reproducible measurements, such as the femoral neck-shaft angle, lateral and vertical center-edge angles, and acetabular roof angle, which is a major step toward generating automated measurements for clinical cases [28, 29].

#### Cross-sectional imaging

CT measurements of the hip are useful for precise evaluation of acetabular morphology, such as acetabular version and depth. Multiplanar reformatted imaging can also be used to assess lateral and anterior center-edge angles along with the maximal femoral head-neck junction alpha angle based on radial imaging of the femoral neck. Supine axial CT should be performed with the hips extended and thighs horizontal and parallel. Care should be taken to align the pelvis so that its transverse long axis is parallel to the table, to eliminate pelvic rotation as much as possible though newer reconstruction techniques can correct this in postprocessing. As with radiography, it should be remembered that supine imaging can underestimate acetabular version angle measurement, and low radiation-dose, standing, weightbearing hip CT techniques, often using cone-beam technology, are gaining more widespread use clinically [30]. Additionally, there is emerging data suggesting three-dimensional reformatted CT images may be useful to standardize measurements by reducing errors due to positional variations. Finally, automated measurement tools are being developed to make measurements more efficient and reproducible. Similar to CT, MRI can be used to evaluate acetabular morphology with the added benefit of identifying labral and chondral abnormalities.

### Measurements

Non-arthritic hip pain requires careful radiographic analysis to evaluate potential biomechanical causes of pain. In general, patients can be categorized into hip instability or hip impingement categories. Hip dysplasia occurs when the acetabulum does not cover the femoral head sufficiently, resulting in hip instability and pain. Instability can also occur with increased femoral version and valgus neck-shaft angles. Hip impingement results from excessive contact between the acetabulum and femoral head-neck junction, also known as femoroacetabular impingement (FAI). FAI commonly consists of both acetabular and proximal femoral deformities. Acetabular over-coverage, retroversion and prominent anterior inferior iliac spine morphology can predispose to impingement. Similarly, insufficient femoral head-neck offset, such as a cam deformity, short varus femoral neck, and femoral retroversion can contribute on the femoral side.

There are multiple useful measurements of the acetabulum and femoral head and their relationships with the pelvis and femur (Table 2).

**Table 2:** Commonly used measurements in the radiologic assessment of the hip

Radiologic measurement	Definition	Imaging modality of choice	Normal value(s)/normal range	Abnormal value(s)/clinical significance
Lateral center-edge angle	Angle formed between a vertical line, and a line from femoral head center to the lateral acetabular roof margin (Fig. 4)	Orthograde AP pelvis or hip radiographs	25º–40º	**·** < 20° acetabular dysplasia **·**20º–25º borderline **·** > 40° probable pincer-type FAI
FEAR index	Angle formed by line connecting the medial and lateral margins of the acetabular roof and a line connecting the medial and lateral borders of the central third of the femoral physeal scar (Fig. 5)	AP hip radiograph	−1.3º to −5°	**·** < −5° associated with FAI **·** > −1.3° assoc. with dysplasia
Acetabular inclination angle	Angle formed between a horizontal line and a line connecting the medial and lateral acetabular roof margins (Fig. 6)	Orthograde AP pelvis radiograph	0º–10º	**·** < 0° possible pincer-type FAI **·** > 10° acetabular dysplasia
Acetabular wall index	Distance along a line drawn down the long axis of the femoral neck between the anterior or posterior acetabular wall and where it intercepts the femoral head best fit circle divided by the radius (Fig. 7)	AP hip radiograph	**·** ~ 0.41 anterior **·** ~ 0.91 posterior **·** > 0.8 anterior/posterior ratio	**·**Lower values seen in dysplasia **·**Higher values seen in deep acetabula **·** < 0.8 A/P ratio suggests retroversion
Acetabular version angle	Angle formed by a line connecting the anterior and posterior acetabular rims, and a line perpendicular to the pelvic transverse long axis (Fig. 8)	Axial CT of hips with coronal reformatted CT image for clock face	11º–23°	**·** < 0° retroverted **·** > 23° anteverted
Acetabular sector angle	Angle formed by a line connecting femoral head centers, and a line from femoral head center to the anterior or posterior acetabular lip. Horizontal sector angle is the sum of the anterior and posterior sector angles (Fig. 10)	Axial CT of hips with coronal reformatted CT image for clock face	**·**Anterior > 50° **·**Posterior > 90° **·**Horizontal > 140°	Indicates dysplasia: **·**Anterior ≤ 50° **·**Posterior ≤ 90° **·**Horizontal ≤ 140°
Anterior center-edge angle	Angle formed between a vertical line and a line from femoral head center to the anterosuperior acetabular roof margin (Fig. 11)	False profile radiograph of the hip	25º–40°	**·** < 20° acetabular dysplasia **·**20º–25° borderline **·** > 45° probable pincer-type FAI
Acetabular angle of Sharp	Angle formed between a horizontal line and a line connecting the acetabular teardrop to the lateral acetabular roof margin (Fig. 12)	Orthograde AP pelvis or hip radiograph	33º–38º	**·**39º–42° borderline **·** > 42° dysplastic
Acetabular depth:width ratio (ADR)	Distance from the teardrop to the lateral acetabular margin divided by the length of a perpendicular line from the depth line to the deepest part of the acetabulum (Fig. 13)	Orthograde AP pelvis or hip radiograph	~ 0.53	< 0.38 can predict development of early osteoarthritis
Femoral inclination angle	Angle formed by lines along the femoral neck and femoral shaft long axes (Fig. 14)	AP hip radiograph	125º–135°	**·** < 125° coxa vara **·** > 135° coxa valga
Alpha angle	Angle formed by a line along the femoral neck long axis, and a line from femoral head center to where the femoral head cortex extends beyond a best fit circle of the femoral head (Fig. 15)	Radial MR or reformatted radial CT images through femoral neck	< 50º–60° depending on institutional threshold	> 50º–60° cam deformity depending on institutional threshold
Femoral head extrusion index	Transverse width of the lateral femoral head not covered by the acetabulum divided by the transverse width of the entire femoral head × 100 (Fig. 16)	AP orthograde pelvic radiograph	17–27% normal	**·** < 17% possible pincer-type FAI **·** > 27% acetabular dysplasia
Ischiofemoral distance	Shortest distance between the lateral cortex of the ischial tuberosity and the medial cortex of the lesser trochanter (Fig. 17)	Axial MRI but can be assessed on AP standing hip XR	≥ 15 mm on CT/MRI ≥ 19 mm on standing XR	**·** < 15 mm possible impingement on CT/MR **·** < 19 mm possible impingement on standing XR
Quadratus femoris distance	Shortest distance between the posteromedial margin of the iliopsoas tendon or lesser trochanter and the superolateral margin of the hamstring tendons (Fig. 17)	Axial MRI	≥ 10 mm on CT/MRI	**·** < 10 mm possible impingement on CT/MR

#### Acetabular coverage measurements

Acetabular-sided measurements are designed to quantify femoral head coverage and determine if coverage is normal, deficient, or excessive, as well as evaluate for versional abnormalities. This can occur with traditional anterolateral deficiency, isolated anterior deficiency, or global retroversion.

##### Lateral center-edge angle

The lateral center-edge angle (LCEA), or the angle of Wiberg, is used to assess the superolateral coverage of the femoral head by the acetabulum. This measurement is most often used as an initial screening tool in determining if acetabular coverage is sufficient.

**Fig. 4:**
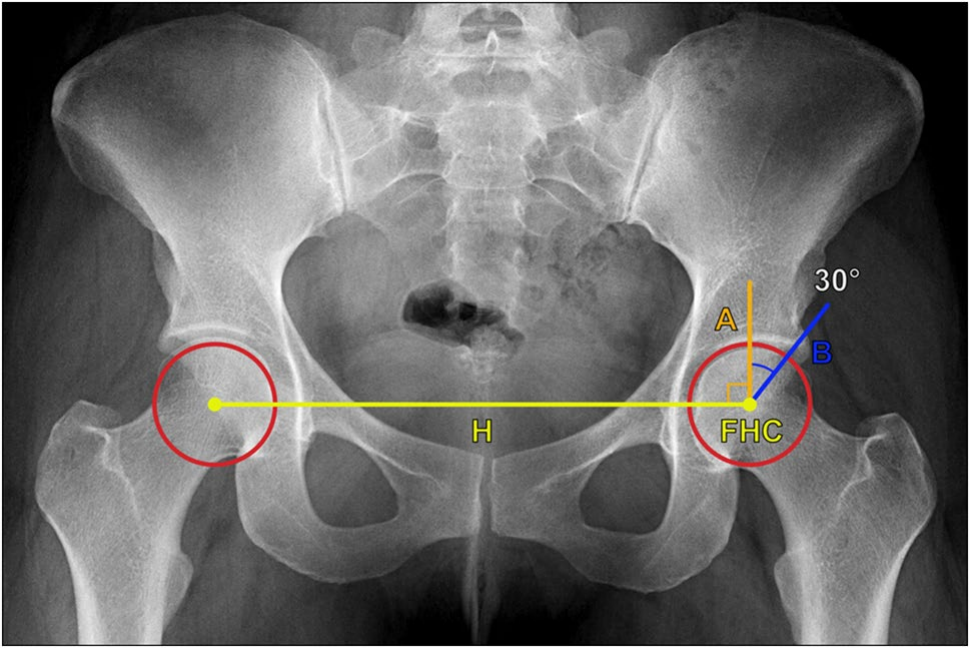
Measurement of the lateral center-edge angle on AP radiograph of the hip. A best-fit circle is drawn over the femoral head. The angle is formed between a line (A, orange line) extending vertically from the center of the circle (FHC), perpendicular to a line connecting the femoral head centers (H, yellow line), and a line (B, blue line) extending from femoral head center to the lateral margin of the superior acetabulum. The normal value is 25º–40°, while values of 20º–25° are borderline and values < 20° indicate acetabular dysplasia

Since abnormal pelvic tilt or rotation can affect the accuracy of this measurement, it is critical to measure the LCEA on true orthograde AP pelvis or hip radiographs. The LCEA is formed by two lines through a best-fit circle of the femoral head: a reference vertical line through the center of the circle and a line extending from the center of the circle to the lateral margin of the acetabular roof (Fig. 4) [26]. To correct for potential differences in patient positioning/pelvic tilt, the vertical line is perpendicular to the short axis of the patient, which is formed by a horizontal line connecting either the ischial tuberosities or the centers of best fit circles of the femoral heads in adults or the Hilgenreiner line connecting the triradiate cartilages in pediatric patients on AP pelvic radiographs [31]. Including superior acetabular rim osteophytes or even developmental spurring results in a larger measured angle, which may result in over-diagnosis of pincer-type FAI. As a result, many authors have suggested excluding these osseous protuberances and using the lateral margin of the acetabular roof as the lateral margin for measurement [32]. When measured on CT or MRI, true coronal CT reformatted images or MR images with the lower extremities in 15º–20° of internal rotation should be obtained to match the radiographic appearance, which enhances measurement standardization and accuracy and decreases interobserver variability. This should be performed at the 12 o’clock position on coronal images. Measurements performed on posterior coronal slices correlate poorly with radiographic measurements [33].

The LCEA normally ranges between 25° and 40°. Values between 20° and 25° represent borderline dysplasia, and those < 20° indicate acetabular dysplasia. On the other hand, measured values > 40° may indicate acetabular over-coverage in the setting of pincer-type FAI [26, 34].

##### Femoro-Epiphyseal Acetabular Roof (FEAR) index

Classification of the borderline dysplastic hip is a controversial topic in adult hip-preservation surgery. In patients with a borderline LCEA, it can be unclear whether the hip should be categorized as unstable or as having impingement; this distinction is important for determining appropriate surgical intervention in patients failing physical therapy. Further, clinically there is a need to identify borderline hips that will behave as stable or unstable. This relatively new concept is known as the FEAR index [35]. A measurement of the angle formed between the physeal scar of the femoral head growth plate and the acetabular roof corresponds to the force vector of the hip with a laterally directed vector potentiating instability and a medially directed vector associated with impingement.

**Fig. 5:**
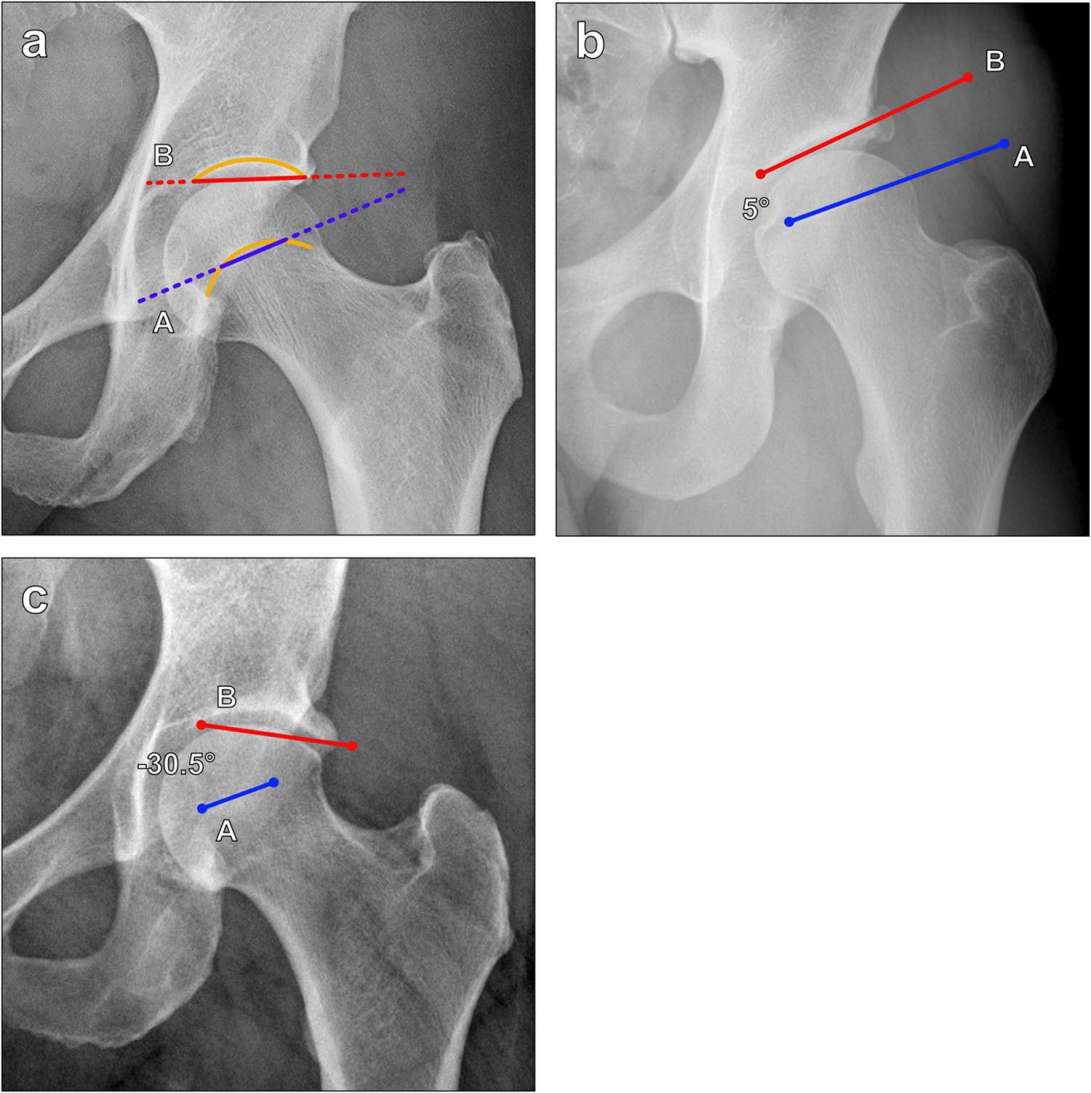
Measurement of Femoro-Epiphyseal Acetabular Roof (FEAR) index on AP hip radiograph. **a** The angle is formed between a line connecting the medial and lateral points of the central third of the femoral head physeal scar (A, blue line) and a line connecting the medial and lateral margins of the acetabular roof (B, red line). A FEAR index > −1.3° is associated with hip dysplasia and a FEAR index < −5° is associated with FAI. **b** AP radiograph of the left hip in 24-year-old male patient with hip pain. In this example, the FEAR index opens laterally and measures 5°, consistent with dysplasia. **c** AP radiograph of the left hip in a 37-year-old female patient with hip pain demonstrates an angle that opens medially and measures −30.5°, consistent with acetabular overcoverage and FAI

Measured on AP hip radiographs, first the central third of the femoral head physeal scar is identified and a straight line is drawn connecting the medial and lateral points of this relatively straight section of the scar. The second line connects the medial and lateral margins of the acetabular roof. The angle formed between these two lines is considered positive if the angle opens laterally, and negative if the angle opens medially (Fig. 5) [35].

In normal and stable borderline hips, the FEAR index is slightly negative (opens medially). More negative values are seen in patients with impingement, and more positive values are seen in patients with dysplasia. A FEAR index more greatly positive than −1.3° is associated with a dysplastic hip, and a FEAR index more greatly negative than −5° is associated with FAI [36].

##### Acetabular inclination angle

The acetabular inclination angle, also known as the horizontal toit externe (HTE) angle or Tönnis angle, is a measure of the steepness of the acetabular roof [37]. Normally, the acetabular roof is parallel or nearly parallel to the horizontal axis of the body, and acetabular dysplasia results in a steepening of the acetabular roof.

**Fig. 6:**
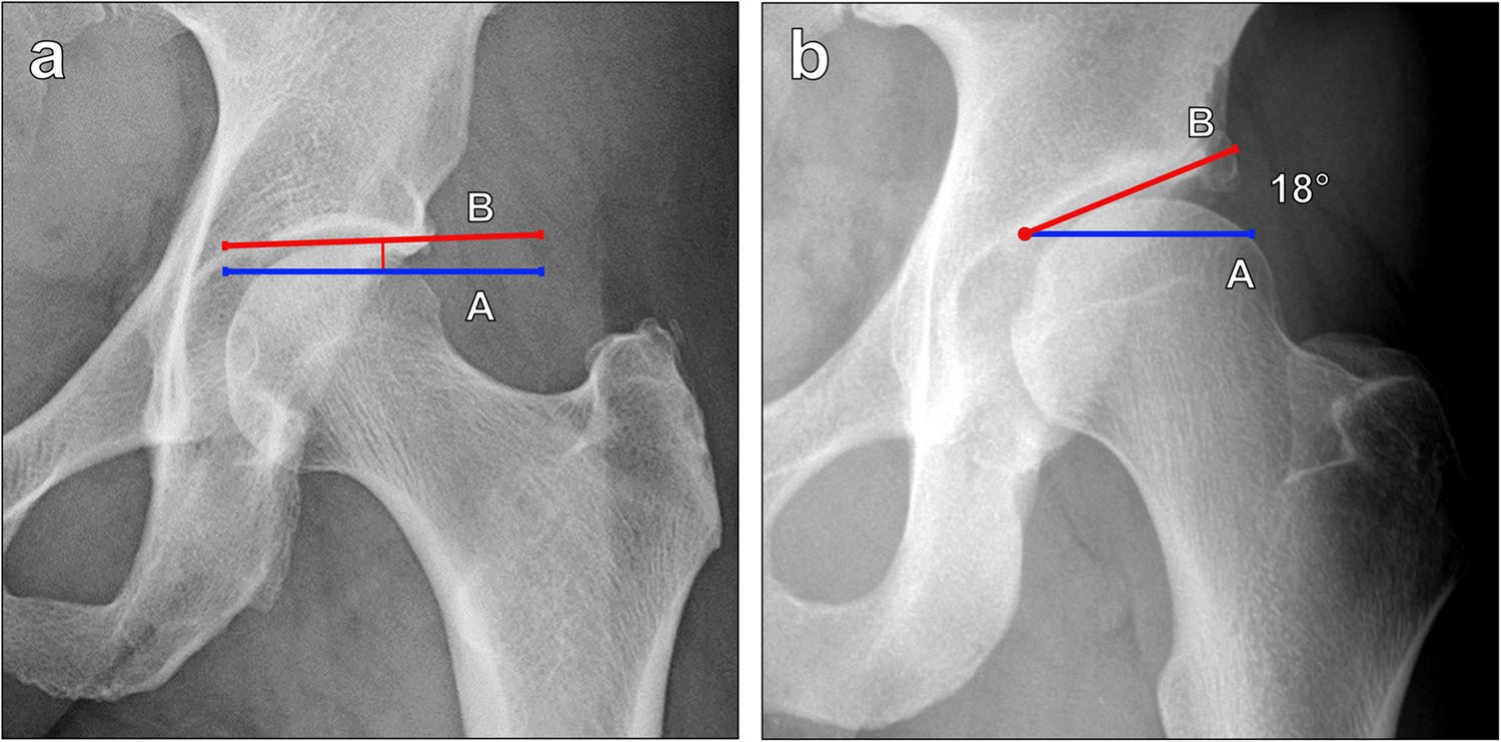
Measurement of acetabular inclination angle on AP radiograph of the pelvis. **a** The image is coned down to the hip to better illustrate the angle measurement. The angle is formed between a horizontal line (A, blue line) and a line connecting the medial and lateral margins of the acetabular roof (B, red line). The horizontal line is formed by connecting the ischial tuberosities or a line bisecting the femoral heads on an orthograde AP pelvic radiograph. The normal measurement values range between 0º and 10°. Values > 10° indicate acetabular dysplasia, and values < 0° are at risk for pincer-type FAI. **b** AP radiograph of the pelvis coned to the left hip and ischial tuberosities in a 24-year-old male with hip pain shows a steepened acetabular inclination angle, measuring 18°, consistent with acetabular dysplasia. The blue line, A, represents the bi-ischial line that indicates the horizontal plane, while the red line, B, connects the medial and lateral margins of the acetabular roof that excludes an os acetabulare

Measured on AP pelvis radiographs, the angle is formed by (1) a horizontal line connecting either the inferior margins of acetabular teardrops, ischial tuberosities, or centers of best-fit circles to femoral heads and (2) a line connecting the medial and lateral margins of the acetabular roof (Fig. 6). When the medial margin of the roof is difficult to see, a modified Tönnis angle, which uses a horizontal line through the apex of the femoral head and a line connecting the medial margin of the horizontal line as it intersects with the acetabular roof and the lateral margin of the acetabular roof, can be used [38].

The angle should measure <30° at birth and become progressively shallower over time, measuring <22° after age 1 [39]. In adults, the normal range of measurement values is between 0° and 10°. Values >10° indicate acetabular dysplasia, and values <0° are at risk for pincer-type FAI [26, 27].

##### Acetabular wall indices (anterior and posterior)

While the LCEA defines the amount of lateral femoral head coverage, the acetabular wall indices are a surrogate for anterior and posterior femoral head coverage.

**Fig. 7:**
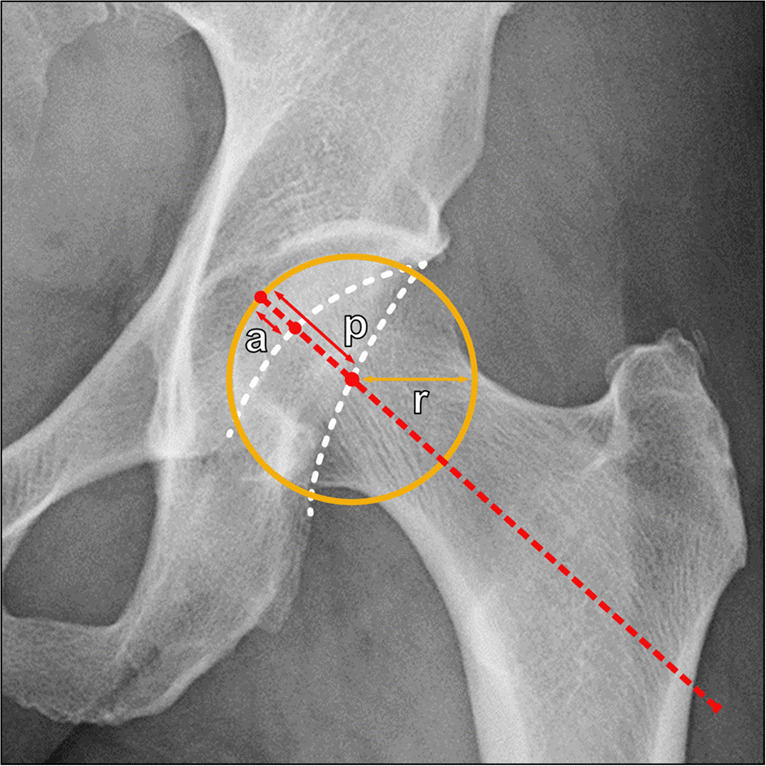
Measurement of acetabular wall indices on AP hip radiograph. A best-fit circle is drawn over the femoral head and the radius of the circle (*r*) is determined. Then a line is drawn down the long axis of the femoral neck intersecting the circle through its center (dashed line). The anterior wall index (AWI) is calculated as the distance along this line between where it intersects the anterior acetabular wall and where it intersects the femoral head best-fit circle medially (*a*), divided by the circle radius (*r*) (AWI = *a*/*r*). The posterior wall index (PWI) is calculated in a similar fashion, with (*p*) representing the distance along the line indicating the femoral neck (dashed line) between the posterior margin of the acetabulum and the point where the line leaves the best-fit circle (PWI = *p*/*r*). The normal AWI measures approximately 0.41, while the PWI measures approximately 0.91. An AWI/PWI ratio < 0.8 suggests acetabular retroversion

Measured on AP hip radiographs, a best-fit circle is drawn over the femoral head, and the radius of the circle is determined. Then a line is drawn down the long axis of the femoral neck intersecting the circle through its center. Note is made of where this line contacts the circle medially, the anterior acetabular wall, and the posterior acetabular wall. The anterior wall index (AWI) is calculated as the distance along this line between where it intersects the anterior acetabular wall and where it intersects the femoral head best-fit circle medially, divided by the circle radius. The posterior wall index is calculated in a similar fashion (Fig. 7). It is vital to measure these values on true AP hip radiographs since even small degrees of pelvic tilt or rotation can cause significant measurement inaccuracy. Although these measurements are routinely determined on AP hip radiographs, CT can be helpful to standardize patient positioning and reduce position-related measurement error associated with radiography.

The average AWI and PWI values in normal hips are approximately 0.41 and 0.91 respectively with lower values seen in dysplastic hips and higher values seen in deep acetabula [40]. An AWI/PWI ratio < 0.8 suggests acetabular retroversion.

##### Acetabular version

Acetabular version is another angular measurement that can help determine the degree of acetabular coverage of the femoral head; however, it assesses the degree of anterior and posterior coverage rather than the superior coverage. There is a normal mild anterior acetabular undercoverage compared with the posterior acetabulum, called anteversion. Excessive anteversion indicates acetabular dysplasia, while neutral version, in which the anterior and posterior acetabular rims equally cover the femoral head, and retroversion, in which the anterior acetabular rim overcovers the femoral head relative to the posterior acetabulum, can contribute to pincer-type FAI.

**Fig. 8:**
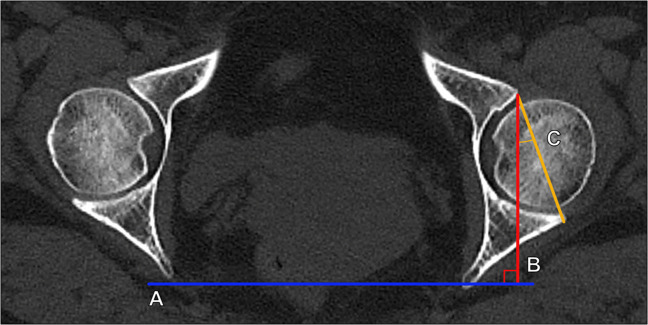
CT evaluation of acetabular version angle. A line connecting the ischial tuberosities serves as the transverse long axis line (A, blue line). The acetabular version angle is formed between a line perpendicular to the transverse long axis (B, red line) and a line connecting the anterior and posterior acetabular rims (C, yellow line). The normal range of acetabular version angles in adults is 11º–23°. Angles > 23° are therefore considered excessively anteverted, while angles < 11° can be associated with pincer-type FAI. 0° is considered neutral version, and values < 0° are considered retroverted

Acetabular version is measured on supine axial CT of the lower pelvis, and both the global/central version and cranial version can be measured. The technologist should make an effort to minimize pelvic tilt to either side, and if necessary, the CT data should be reformatted to create true axial images of the pelvis with the inferior margin of the ischial tuberosities located on the same axial CT image in most patients. Measurement of the acetabular version angle also requires a coronal CT reformatted image of the hip at the apex of the femoral head to establish a clock-face for localization. On the coronal reformatted CT image, a best-fit circle can be drawn using the acetabular roof (or the femoral head if it is centered in the acetabulum as a proxy); the most superior point is the 12 o’clock position, while the 6 o’clock position is the most inferior point of the circle and 3 o’clock is lateral at the acetabular equator. The equator is the horizontal diameter of the circle. Global/central acetabular version should be measured on the axial CT image located at the acetabular equator, while cranial acetabular version should be measured on the axial CT at the 1:30 position on the clock face. To form the acetabular version angle, the transverse long axis of the pelvis must first be established by creating a line connecting the ischial tuberosities. The acetabular angle is formed by a line perpendicular to the transverse long axis and a line connecting the anterior and posterior acetabular rims (Fig. 8) [41].

Acetabular anteversion at birth is generally low and increases during childhood until skeletal maturity. The normal range of acetabular version in adults is 11º–23° [42]. Angles > 23° are therefore considered excessively anteverted, 0° is considered neutral version, and values < 0° are considered retroverted.

In addition to the acetabular version measurements, qualitative analysis generally includes the crossover sign, crossover index, posterior wall sign, and ischial spine signs. Lastly, Tönnis classification is most commonly used for arthritic grading in the hip [43].

##### Increased acetabular depth

**Fig. 9:**
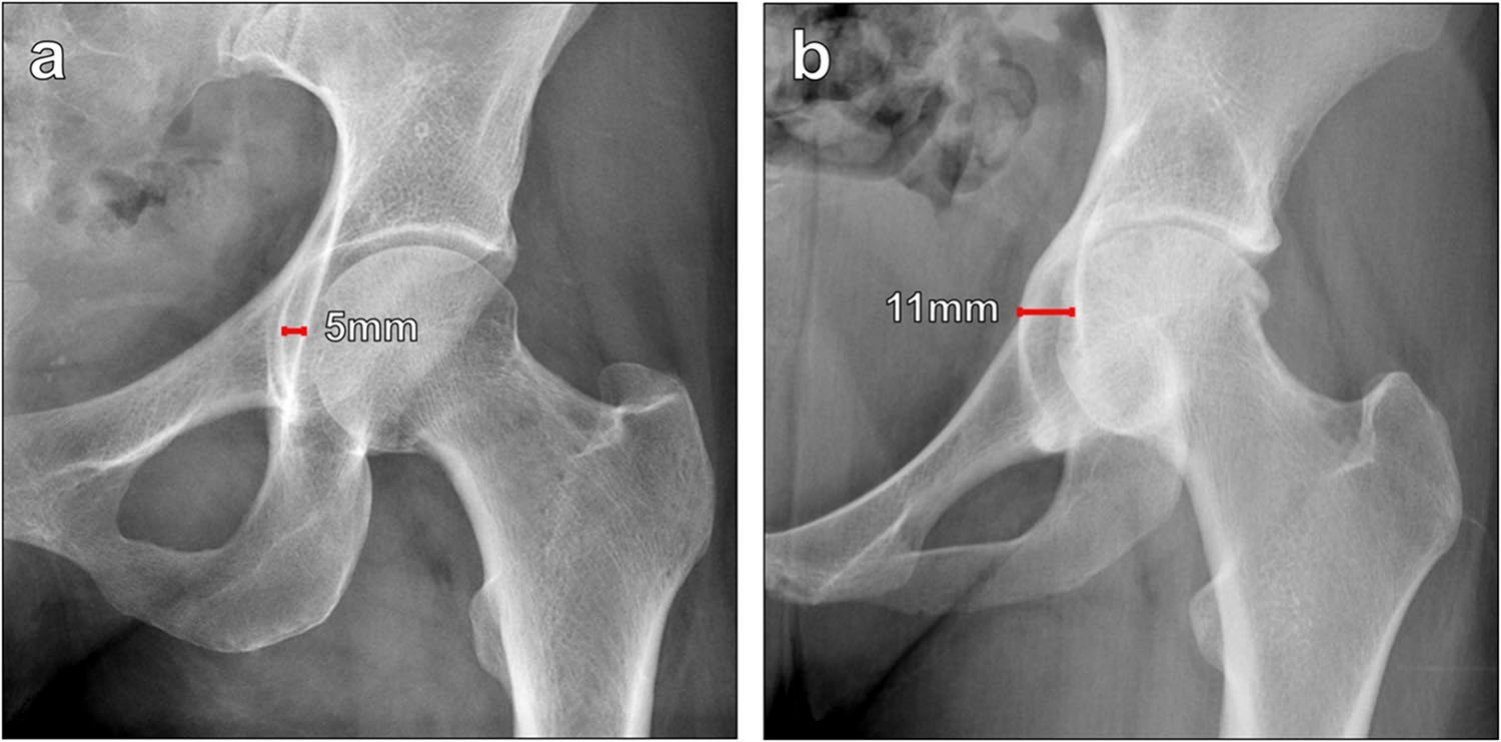
Deep acetabula on AP pelvic radiographs. **a** Coxa profunda in a 47-year-old female patient with mild hip pain. This can be diagnosed radiographically when the medial acetabular wall either overlaps with the ilioischial line on a true orthograde AP pelvic radiograph or is located slightly medial to it. This can be incidental or associated with pincer-type femoroacetabular impingement. **b** Protrusio acetabuli in a 24-year-old female patient with hip pain. The distance between the lateral margin of the teardrop and the ilioischial line measures 11 mm, and the medial cortex of the acetabulum protrudes slightly into the pelvis. Protrusio acetabuli is a more severe deepening of the acetabular fossa and can be diagnosed on true orthograde AP pelvic radiographs in which the lateral border of the teardrop is located > 3 mm medial to the ilioischial line in men and > 6 mm in women. This results in femoral head overcoverage and can be associated with femoroacetabular dysplasia

A deep acetabular fossa can be associated with femoral head overcoverage and hip pain. On orthograde AP pelvic radiographs, normally, the medial acetabular wall, called the teardrop radiographically, should be lateral to the ilioischial line. With milder forms of acetabular fossa deepening, called *coxa profunda*, the lateral border of the teardrop either overlaps or is located slightly medial to the ilioischial line, and measures ≤ 3 mm in men and ≤ 6 mm in women (Fig. 9a). This can be incidentally noted in asymptomatic patients or associated with pincer-type FAI.

A more severe deepening of the acetabular fossa is *protrusio acetabuli*, in which the medial acetabulum and femoral head protrude into the pelvis. This is diagnosed when the lateral cortex of the teardrop is located >3 mm medial to the ilioischial line in men or >6 mm in women (Fig. 9b). Care should be taken to measure this only on true orthograde AP pelvic radiographs, since any obliquity can either obscure or artificially produce this finding. Acetabular protrusion can be idiopathic or associated with a variety of conditions, such as OA, rheumatoid arthritis, Paget’s disease of the bone, or hyperparathyroidism. Greater degrees of acetabular deepening can be associated with pincer-type FAI with acetabular labral tear and chondral loss.

#### Less commonly used acetabular coverage measurements

While some clinicians may request the following measurements, these are less frequently utilized in the literature. Proper measurement technique is included in the corresponding figures, and normal values for each are reported in Table 2.

##### Acetabular sector angle

**Fig. 10:**
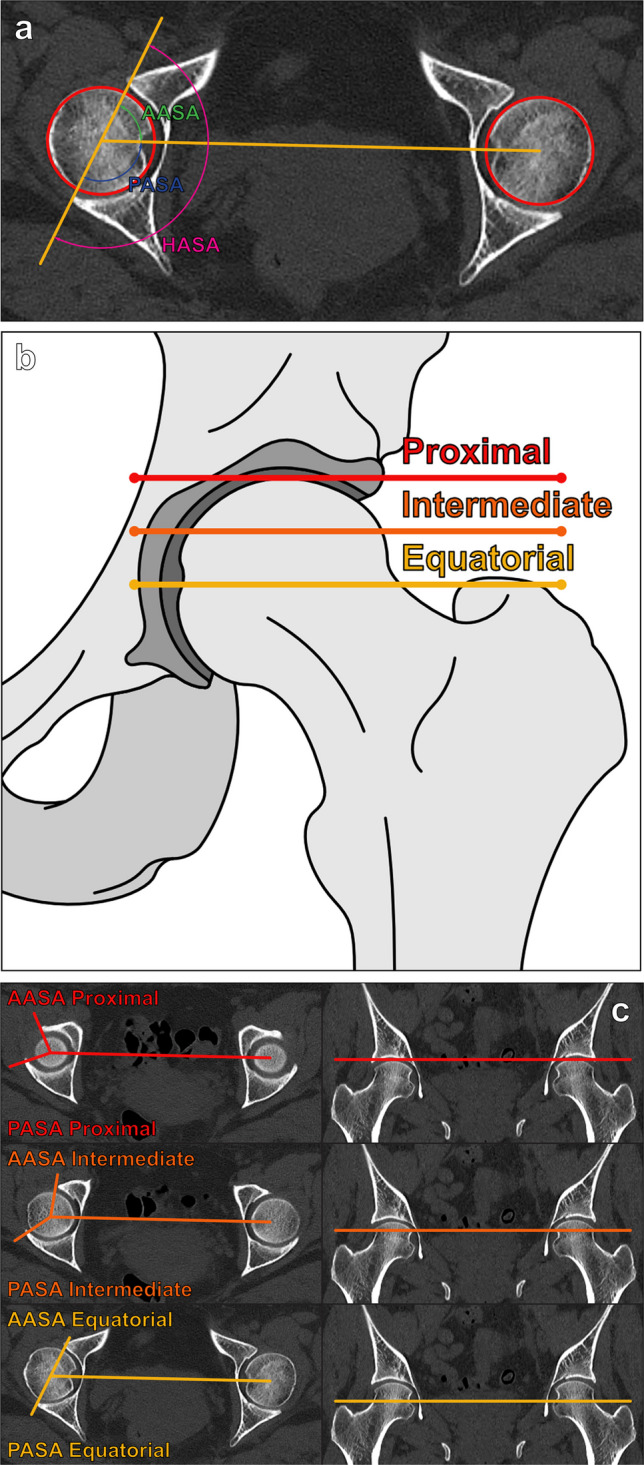
Measurement of the acetabular sector angle on axial CT image located at the femoral head center. First, a line is drawn connecting the center of a best-fit circles of the femoral heads. Next, lines are drawn from the center of the femoral head to the anterior and posterior lips of the acetabulum forming the anterior (AASA, green arc) and posterior (PASA, blue arc) acetabular sector angles, respectively. Adding these two angles together produces the horizontal acetabular sector angle (HASA, pink arc) which evaluates for global deficiency of femoral head coverage. Using this method, an AASA of > 50°, a PASA of > 90°, and a HASA > 140° is considered adequate acetabular coverage. **b** Frontal diagram of the hip depicts the axial planes of section for the three-level assessment of acetabular sector angles at the 12:00 (red line), 1:30 (orange line), and 3:00 (yellow line) positions. **c** Axial and coronal reformatted imaging of the hips demonstrating the three-level assessment shown in part **b** along with how the angles can change from one level to the next. Proposed cutoff values for acetabular dysplasia with the more detailed method are as follows: proximal AASA < 133°, equatorial AASA < 57°, proximal PASA < 137°, intermediate PASA < 102°

Morphologic analysis of acetabular deficiencies can be performed by measuring the anterior and posterior acetabular sector angles (Fig. 10). These angles can be helpful to assess regional areas of acetabular dysplasia, often for preoperative planning [44]. The anterior acetabular sector angle (AASA) and posterior acetabular sector angle (PASA) quantify the degrees of anterior and posterior acetabular coverage respectively, while the horizontal acetabular sector angle (HASA) assesses the degree of acetabular coverage globally. Using this method, an AASA of > 50°, a PASA of > 90°, and a HASA > 140° is considered adequate acetabular coverage. Smaller AASA and PASA values suggest decreased anterior or posterior acetabular coverage and localized dysplasia respectively.

Some clinicians have requested this analysis at three separate axial CT levels (Fig. 10c) to determine how the degree of coverage varies from cranial to caudal. The proximal measurement is obtained at the first axial slice to include the femoral head, the equatorial measurement is at the level of the femoral head center, and the intermediate measurement is the midpoint between these two. These roughly correlate to the 12:00, 1:30, and 3:00 positions, respectively, when the acetabulum is viewed on coronal reformatted images as a clock face.

##### Anterior center-edge angle

**Fig. 11:**
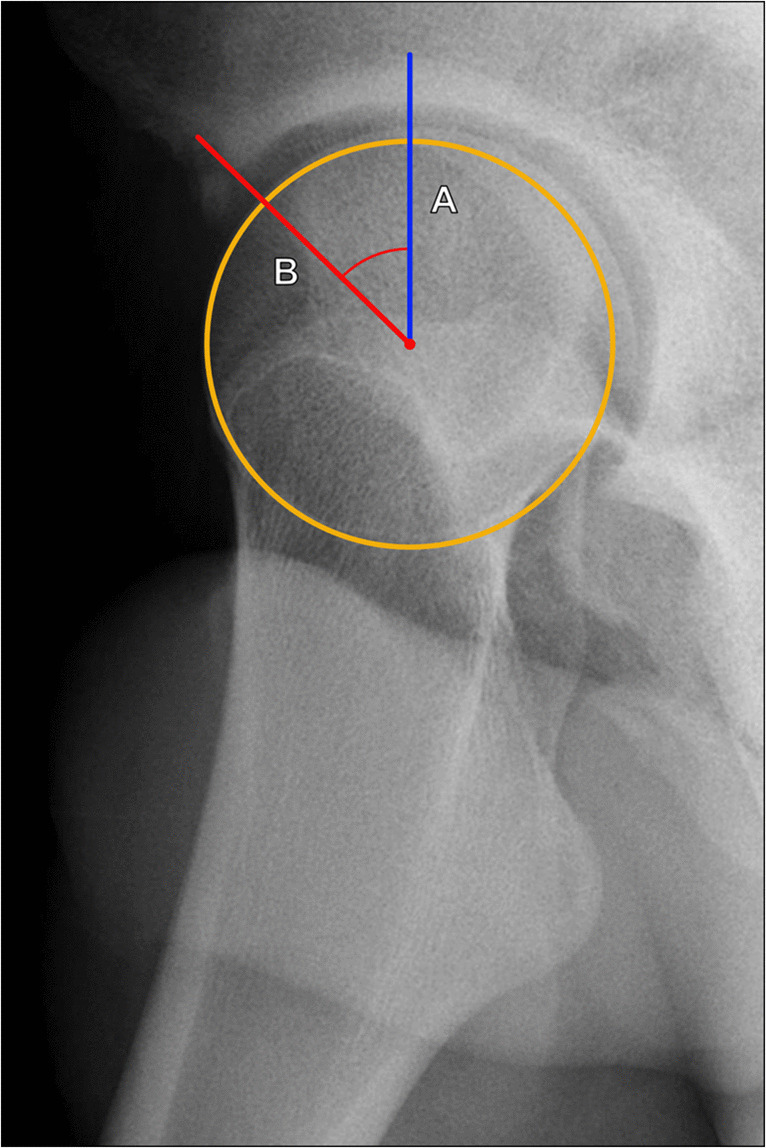
Anterior center-edge angle measurement on false profile radiograph of the hip. A best-fit circle is drawn over the femoral head. The angle is formed between a vertical line (A, blue line) through the center of the best-fit circle and an oblique line (B, red line) from the center of the best-fit circle to the lateral border of the acetabular roof. The ACEA assesses the degree of anterior femoral head coverage and is complementary to the lateral center-edge angle. Normally, the ACEA measures > 25°. Measurements between 20° and 25° are considered borderline, and those < 20° indicate acetabular dysplasia. A measurement > 40º–45° is associated with pincer-type FAI

Measured on false profile radiographs of the hip, the anterior center-edge angle (ACEA), also known as the vertical center-edge angle or angle of Lequesne [45], assesses anterior femoral head coverage and is used as an adjunct to the LCEA in the setting of hip dysplasia (Fig. 11). However, overshadowing by the innominate bone can result in inaccurate measurements of anterior coverage, and anterior and posterior wall indices may be a better reflection of coverage and propensity for anterior or posterior deficiency [46, 47]. The normal ACEA measures 25º–40°. Measurements between 20° and 25° are considered borderline, and those < 20° indicate acetabular dysplasia. A measurement > 40º–45° is associated with pincer-type FAI.

##### Acetabular angle of Sharp

**Fig. 12:**
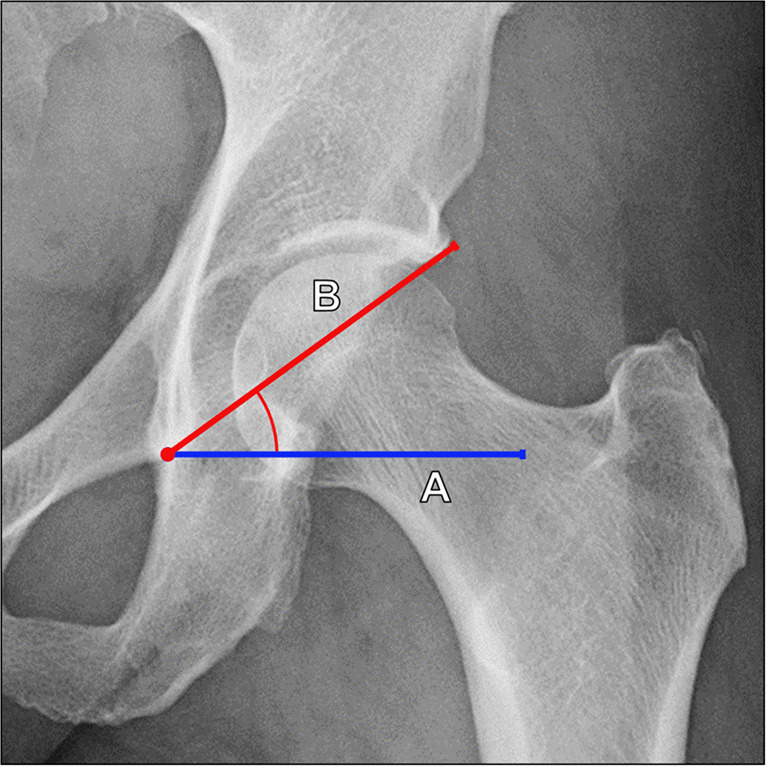
Measurement of the acetabular angle of Sharp on AP hip radiograph. The angle is formed between a horizontal line (A, blue line) and a line (B, red line) connecting the acetabular teardrop to the lateral margin of the acetabular roof. In adults, the normal Sharp angle ranges from 33º to 38°. Angles between 39° and 42° are considered borderline, and angles > 42° are considered dysplastic

The acetabular angle of Sharp (Fig. 12) is another measurement of total acetabular inclination. It is based on the theory that dysplastic hips concentrate force on the weight-bearing acetabular roof, predisposing this location to early cartilage degeneration and development of OA [48, 49]. In adults, the normal Sharp angle ranges from 33º to 38°. Angles between 39° and 42° are considered borderline, and angles >42° are considered dysplastic.

##### Acetabular depth:width ratio

**Fig. 13:**
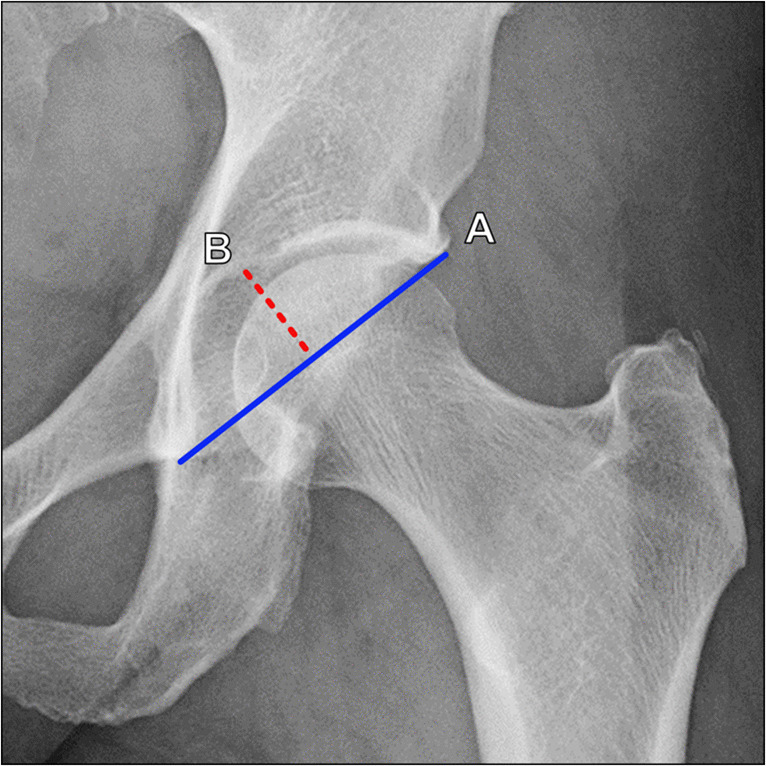
Measurement of the acetabular depth:width ratio (ADR) on an AP radiograph of the hip. The acetabular width (A, blue line) is measured from the inferior margin of the acetabular teardrop to the lateral margin of the acetabular roof, and the acetabular depth (B, red line) is measured as a perpendicular line to the deepest part of the acetabulum. Lower ADR values indicate acetabular dysplasia, while higher values can suggest pincer-type FAI

Because dysplastic acetabula are often wider and shallower than normal acetabula, the ratio of acetabular depth to width, the acetabular depth:width ratio or ADR, is a measure of the degree of dysplasia. (Fig. 13) [50–52]. While the technique for measurement is relatively standard across studies, some authors report raw depth/width values, some multiply by a factor of 100, and others by a factor of 1000, complicating comparison among studies. In normal hips, the ADR raw value is approximately 0.53. A shallow and wide acetabulum will have a low ratio, indicating a greater degree of dysplasia. A value of <0.38 has been found to predict development of OA before 65 years of age.

#### Femoral measurements

##### Femoral inclination angle

**Fig. 14:**
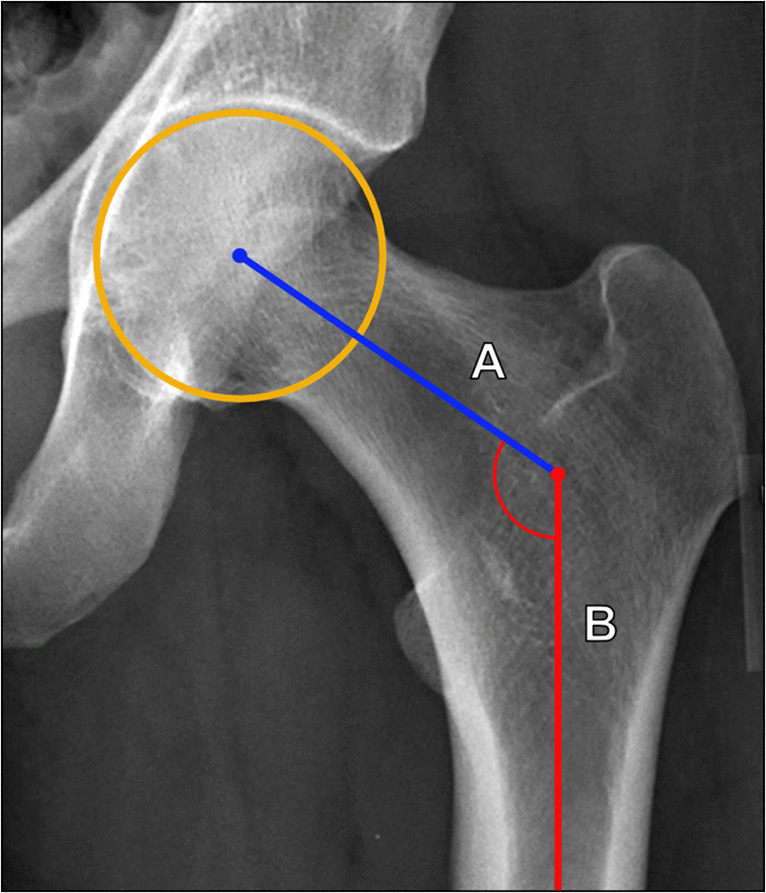
Measurement of the femoral inclination angle (also known as the femoral neck-shaft angle) on an AP radiograph of the hip. A best-fit circle is made over the femoral head. The femoral inclination angle is formed between a line (A, blue line) along the axis of the femoral neck which connects to the center of the best-fit circle, and a line (B, red line) along the axis of the femoral shaft. The normal range is between 125° and 135°, while a measurement >135° indicates coxa valga and a value <125° indicates coxa vara

The femoral inclination angle, also called the collodiaphyseal angle, femoral neck-shaft angle (NSA), or caput-collum-diaphyseal (CCD) angle, is an important anatomic measurement with biomechanical and clinical significance in various orthopedic conditions, including developmental hip dysplasia and OA [53, 54]. It is measured on AP radiographs of the hip or coronal CT or MRI (Fig. 14). A properly aligned true AP view of the hip is mandatory for accurate assessment, as femoral rotation can change measurement, and even slight femoral external rotation can overestimate the angle [55]. First, a best-fit circle is drawn around the femoral head, and a line is drawn from the center of the best-fit circle along the femoral neck axis. A second line is then drawn along the axis of the proximal femoral diaphysis. The femoral inclination angle is the angle formed between the femoral neck and diaphyseal axes [56, 57].

At birth, the femoral inclination angle typically measures 135º–140° [58], and it decreases during normal development. There is slight variation between genders and ethnicities [59], and after skeletal maturity, it measures 125º–135° [60] with a global mean of 129° [61]. Coxa vara is defined as an angle < 125°, while coxa valga is defined as an angle > 135° [26]. Clinically, patients with high femoral inclination angles are at a higher risk for hip instability while low angles increase the risk of impingement.

##### Alpha angle

The alpha angle helps detect asphericity of the proximal femoral head-neck junction, though there are some inherent challenges with its measurement. The generated angle has a high degree of inter-observer variability. Additionally, depending on the position of the offset deformity, it may not be visible on certain radiographs.

**Fig. 15:**
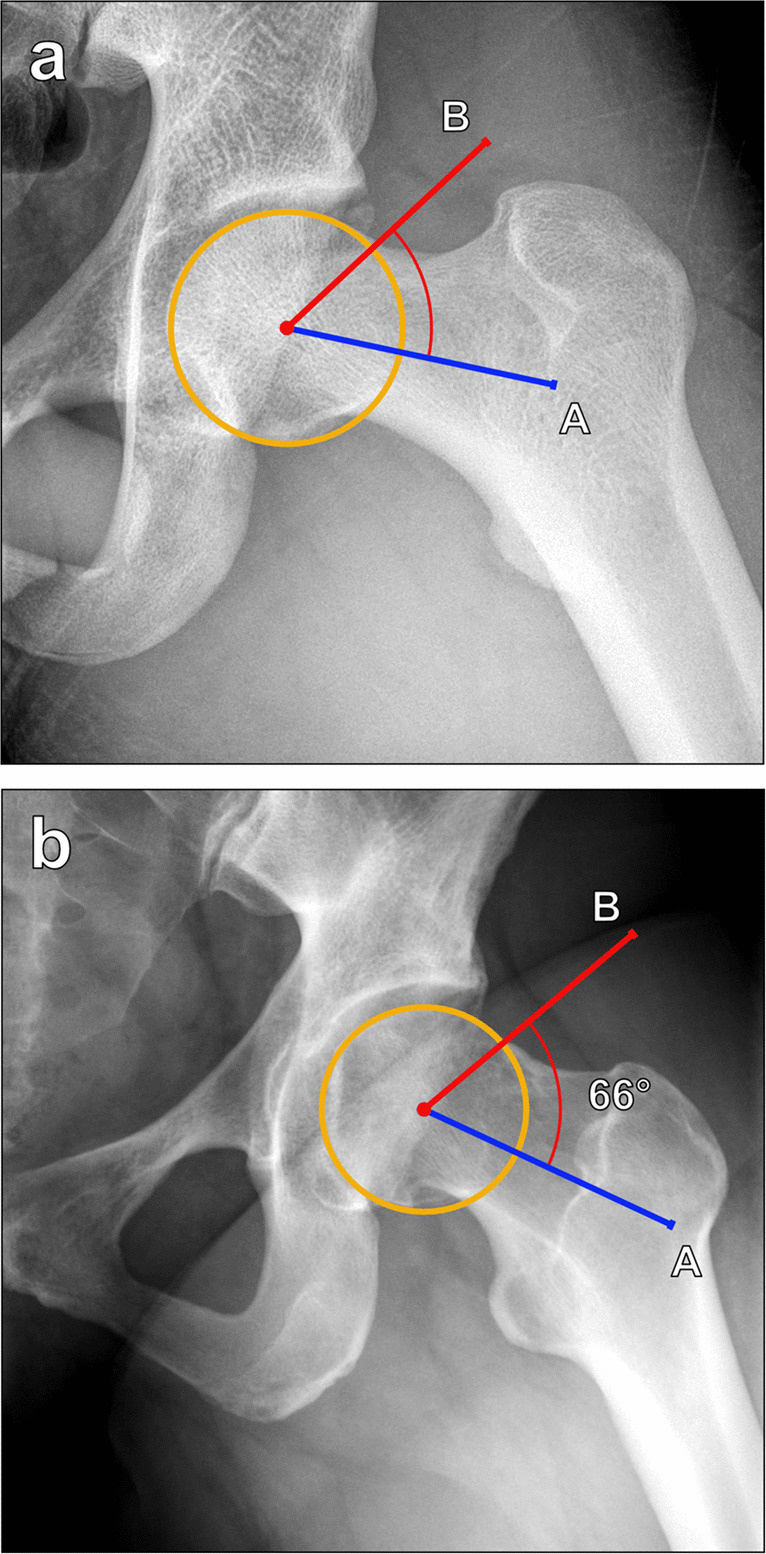
Measurement of the alpha angle on a Dunn radiograph of the hip. **a** A best-fit circle is drawn over the femoral head. The alpha angle is formed between a line (A) from the center of the best-fit circle along the axis of the femoral neck and a line (B) from the center of the best-fit circle to the point of the femoral head/neck junction that extends beyond the circle. The alpha angle is used to detect femoral head asphericity in the setting of cam morphology. Its threshold value is often institution-specific and it ranges from 50º to 60°. Values greater than the institutional threshold suggest a cam lesion. **b** Increased alpha angle indicating cam deformity on a Dunn radiograph in a 52-year-old female patient with left hip pain

The gold standard for assessing cam lesions of the femoral head and neck is on radial MR or reformatted radial CT images along the femoral neck long axis and planned from its short axis. Radiographs can be used clinically as screening tools. The 45° Dunn view hip radiograph is the most sensitive for detecting cam lesions; frog-leg lateral radiographs have been used but may be unreliable [62]. The alpha angle is obtained by drawing two lines from the center of a best-fit circle of the femoral head (Fig. 15). The first line is along the long axis of the femoral neck, and the second line is drawn to the point at which the femoral head-neck cortex extends beyond the best-fit circle [63]. Accepted normal values vary by institution, with a range of 50º–60°. Values greater than the institutional threshold indicate cam morphology [64].

##### Femoral head extrusion index

**Fig. 16:**
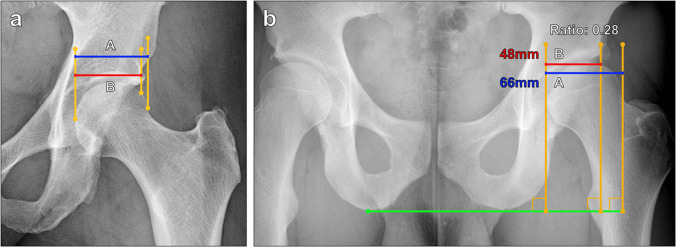
Measurement of the femoral head extrusion index on orthograde AP pelvic radiograph coned to the left hip. **a** Horizontal lines are drawn between the medial and lateral borders of the femoral head (*A*) and between the medial border of the femoral head and the lateral border of the acetabular roof (*B*). The acetabular extrusion index is calculated as [(*A*−*B*)/*A*] × 100%. The normal acetabular extrusion index measures ≤ 27°, and values > 27º–30° indicate acetabular dysplasia. **b** In a 24-year-old male patient with left hip pain, the femoral head extrusion index measures 28%, suggesting borderline acetabular dysplasia

The femoral head extrusion index utilizes a linear measurement to help diagnose acetabular dysplasia. The horizontal distance between the medial and lateral borders of the femoral head and the horizontal distance between the medial border of the femoral head and the lateral border of the acetabular roof are measured on an AP pelvic radiograph (Fig. 16). The percent lateral femoral head extrusion is the transverse width of the lateral femoral head uncovered by the acetabulum divided by the transverse width of the entire femoral head, which is then multiplied by 100 to give a percentage [65]. Normally, this ranges between 17 and 27%. A measurement > 27% indicates acetabular dysplasia, while a value < 17% may indicate pincer-type FAI [66].

#### Ischiofemoral impingement (IFI)

Since the first description of an impingement syndrome between the femoral lesser trochanter and the ischium by Johnson in 1977 [67], ischiofemoral impingement (IFI) has become increasingly recognized as a cause of hip pain, particularly after a more comprehensive study by Torriani et al. in 2009 [68]. Adult females are most commonly affected, presenting with position-dependent buttock or groin pain with occasional clicking/snaping that worsens with hip extension, adduction, and external rotation. This occurs due to repetitive impingement of the quadratus femoris muscle, which passes between the ischial tuberosity and the femoral lesser tuberosity. Any cause of narrowing within this region, such as hip dysplasia, coxa valga, femoral fractures with posttraumatic deformity or even OA leading to medial femoral head migration, can contribute to impingement.

**Fig. 17:**
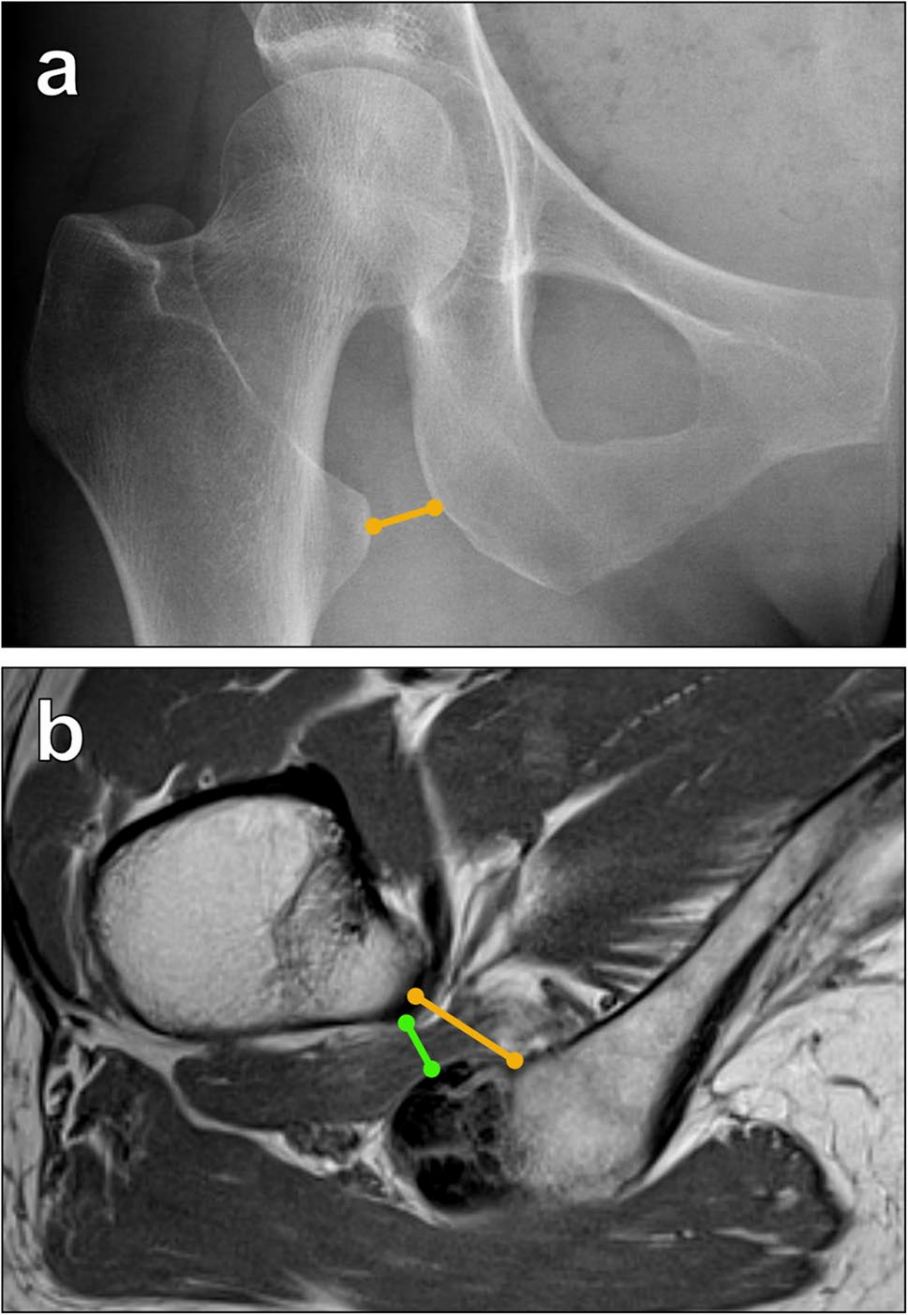
Measurements for ischiofemoral impingement (IFI) on AP hip radiograph (**a**) and axial T1-weighted MR image at the level of the lesser trochanter (**b**). The ischiofemoral distance is defined as the shortest distance between the lateral cortex of the ischial tuberosity and the medial cortex of the lesser trochanter (yellow line). MRI is the preferred imaging modality to make these assessments since it depicts soft tissues, such as the hamstring tendon and quadratus femoris muscle. An ischiofemoral distance < 15 mm on MRI or < 20 mm on radiographs can be associated with IFI. The quadratus femoris distance is defined as the shortest distance between the posteromedial margin of the iliopsoas tendon or lesser trochanter and the superolateral margin of the hamstring tendons (green line). A quadratus femoris distance < 10 mm can be associated with IFI

The measurement of narrowing can be based on osseous landmarks (ischiofemoral distance) or soft tissue landmarks (quadratus femoris distance), and if bony anatomy is used, the distance can be assessed on conventional radiography with AP hip or pelvic radiographs, on axial CT or MR images (Fig. 17). Imaging the entire pelvis including both hips can be helpful to highlight subtle asymmetric cases, though bilateral IFI is reported in up to 40% of cases [69]. MRI is used to assess soft tissue landmarks, such as the hamstring tendon origin and iliopsoas tendon insertion, as well as the status of the quadratus femoris muscle, which is commonly edematous and partially torn or atrophied in this location. There are also cases of patients with quadratus femoris edema or atrophy and ischiofemoral or quadratus femoris space narrowing in the absence of pain. While this may suggest a lack of specificity in these findings, they also raise the possibility for subclinical IFI that has yet to be critically examined [70]. While these measurements are complementary and often used together, the quadratus femoris distance may be more sensitive in detecting signs of impingement since it establishes the actual, functional degree of narrowing and MRI can depict signs of muscle impingement.

##### Ischiofemoral distance

On axial CT or MR images, the ischiofemoral distance is defined as the shortest distance between the lateral cortex of the ischial tuberosity and the medial cortex of the lesser trochanter (Fig. 17b). In normal patients this space measures 18–34 mm, while in patients presenting with IFI, it measures 10–20 mm. Given the overlap of these values, setting a threshold of 15 mm balances specificity and sensitivity, but narrow measurements without secondary findings should be interpreted with caution [70]. Radiographically, an ischiofemoral distance of <19 mm on standing AP hip radiographs and <20 mm on supine AP hip radiographs can be associated with IFI [71].

##### Quadratus femoris distance

The quadratus femoris distance is defined as the shortest distance between the posteromedial margin of the iliopsoas tendon or lesser trochanter and the superolateral margin of the hamstring tendons (Fig. 17b). Because this relies on assessing soft tissue landmarks, MRI is the test of choice to determine this distance. Normally, this measures 10–22 mm, while in patients presenting with impingement, it measures 6–14 mm [70]. Setting a threshold of 10 mm again balances specificity and sensitivity, but a narrow space with no secondary findings of impingement may be normal.

#### Hip arthroplasty

The goals of total hip arthroplasty are to alleviate pain and replicate normal hip biomechanics, including correcting leg length discrepancy, restoring the femoral head center of rotation and total femoral offset. This helps to maintain appropriate range of motion, reduce abnormal prosthetic wear and balance muscle forces between the limbs to provide stability and improve function (Table 3). On postoperative radiographs, the femoral head should be symmetrically seated within the acetabular cup; a superiorly located femoral head indicates polyethylene wear.

**Table 3:** Commonly used measurements in the radiologic assessment of hip arthroplasty

Radiologic measurement	Definition	Imaging modality of choice	Normal value(s)/normal range	Abnormal value(s)/clinical significance
Vertical center of rotation	Distance between the femoral head center and a horizontal line connecting the two ischial tuberosities (Fig. 18)	Weightbearing AP pelvis radiograph	Similar to contralateral hip	Greater vertical center of rotation leads to adductor muscle dysfunction and accelerated prosthetic wear Decreased vertical center of rotation can lead to joint instability and limb lengthening
Horizontal center of rotation	Distance between the femoral head center and the margin of the teardrop (Fig. 18)	Weightbearing AP pelvis radiograph	Equal to contralateral hip	Both increased and decreased horizontal center of rotation can lead to decreased abductor muscle function, gait disturbances and poor mobility
Radiographic lateral acetabular inclination	Angle formed by a line along the acetabular cup rim, and a horizontal line connecting the teardrops (Fig. 18)	AP pelvis or hip radiograph	40° ± 10°	**·** < 30° can result in limited abduction **·** > 50° may increase risk of hip dislocation
Acetabular cup anteversion	Angle formed by a line connecting the anterior and posterior acetabular cup rim and a line perpendicular to the transverse long axis of the pelvis (Fig. 20)	Axial CT	15° ± 10°	**·** > 25° predisposes to anterior hip dislocation **·** < 5° predisposes to reduced range of motion
Acetabular cup inclination	Angle formed by a line along the horizontal axis of the pelvis and a line along the acetabular cup rim (Fig. 20)	Reformatted coronal CT of pelvis or AP pelvis radiograph	40° ± 10°	**·** > 50° predisposes to hip instability/dislocation **·** < 30° predisposes to reduced range of motion

##### Leg length discrepancy

**Fig. 18:**
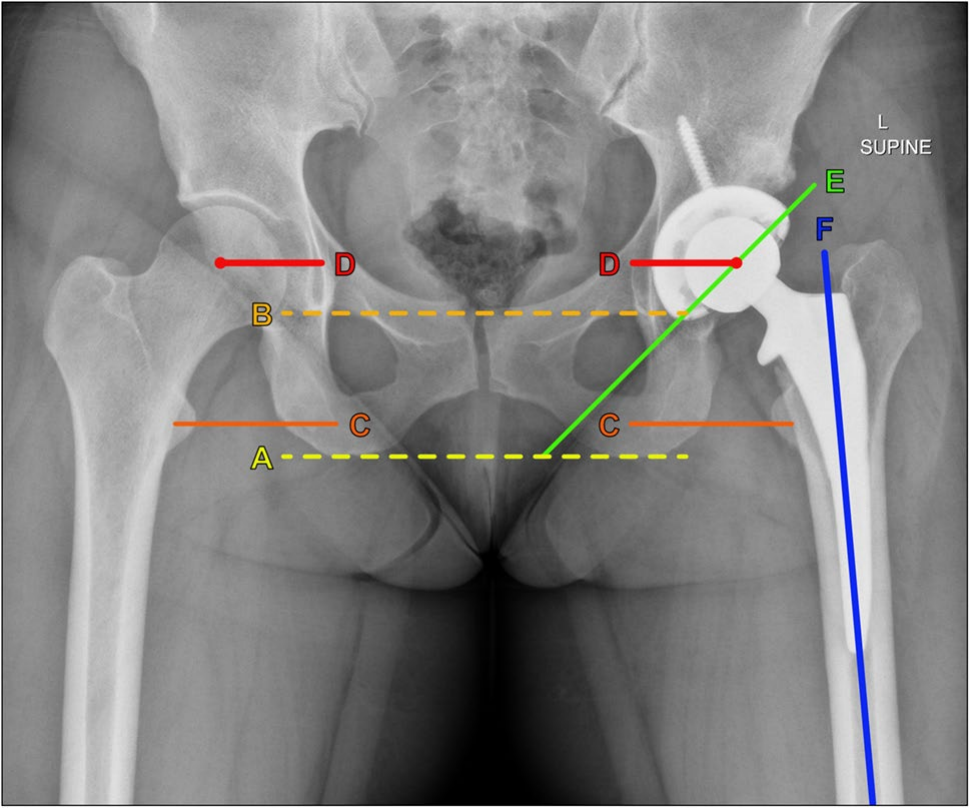
Assessment of total hip arthroplasty component position on AP pelvis radiograph. Line A (dashed lighter yellow line) is a horizontal line connecting the inferior aspects of the ischial tuberosities. Line B (dashed darker yellow line) is a horizontal line connecting the inferior aspects of the acetabular teardrops. The two lines labeled C (solid orange lines) are horizontal lines parallel to the teardrop line through the center of each femoral lesser trochanter. The two lines labeled D (red lines) are horizontal lines from the femoral head center (dot) to the medial teardrop margin. Line E (green line) is a tangential line connecting the medial and lateral edges of the acetabular cup and intersecting line A. Line F (blue line) is a line along the long axis of the femoral stem. Regional limb length is measured as the difference in vertical distance between lines B and C. Acetabular vertical center of rotation is the difference in vertical distance between the femoral head center (dot) and line A. Acetabular horizontal center of rotation is the length of line D. Lateral acetabular inclination is the angle formed between lines E and A

Leg length inequality is common after hip arthroplasty and significantly contributes to patient dissatisfaction following total hip arthroplasty. The total leg length measurement technique is described separately in the limb length section. The degree of leg length discrepancy related to the hip can be measured on AP standing pelvis radiographs, also known as the “regional limb length.” To measure the regional limb length, first a horizontal line connecting the inferior aspects of the acetabular teardrops is drawn, then a parallel line is drawn through the mid-point at the center of the lesser trochanter. The distance between these two lines is the regional limb length measurement (Fig. 18), and the difference in these measurements between the right and left hip is considered the “discrepancy” [72]. A leg length discrepancy < 10 mm is usually well tolerated. Patients may have physiologic variability in anatomy between sides, which is why it is important to compare pre- and postoperative measurements.

##### Femoral offset

**Fig. 19:**
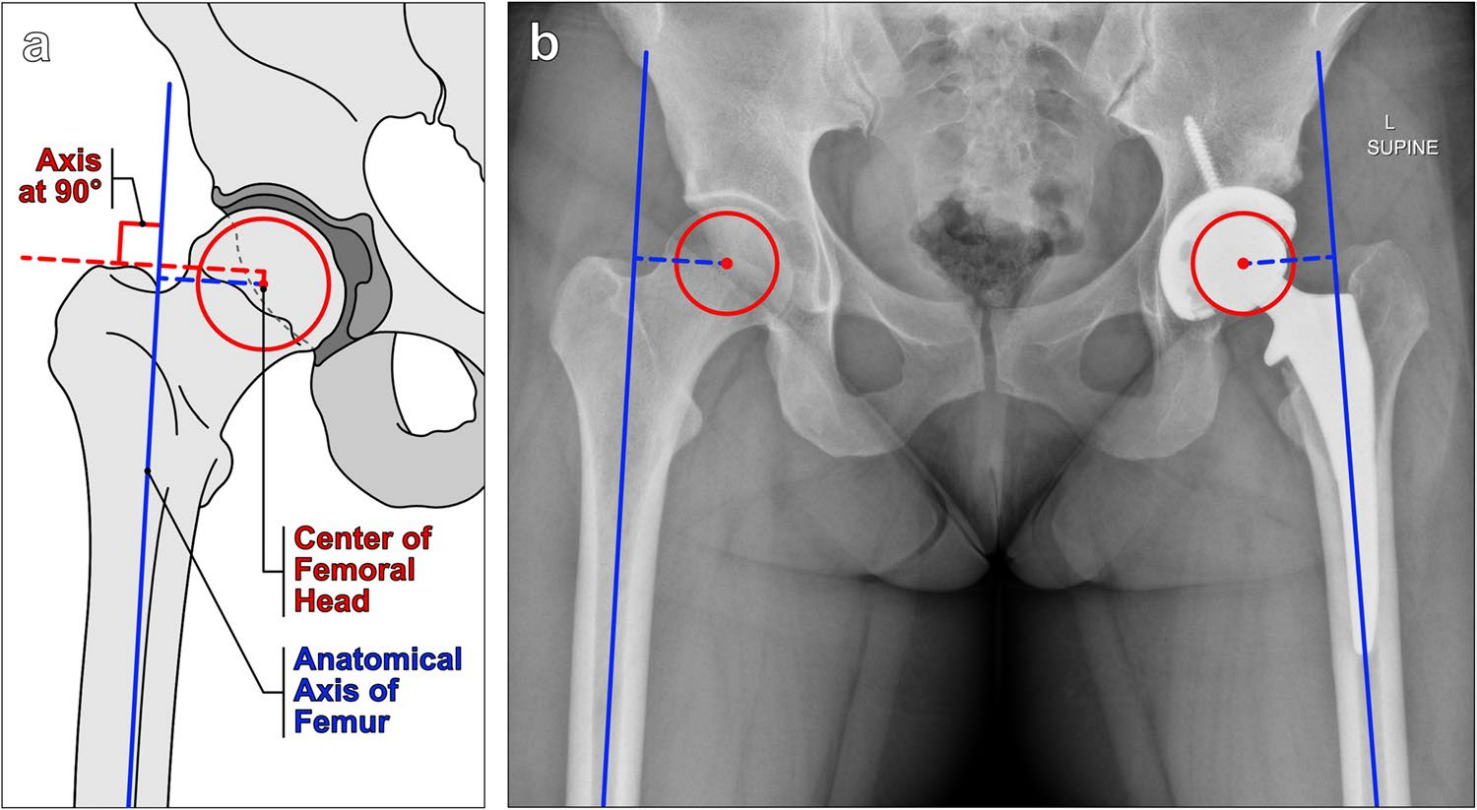
Measurement of femoral offset. **a** Frontal diagram of the hip demonstrates the concept of femoral offset. The center of a best-fit circle involving the femoral head (red dot) is determined, after which the anatomical axis of the femur (solid blue line) is drawn. The femoral offset is defined as the perpendicular distance (dashed blue line) from the femoral head center of rotation to the femoral anatomic axis. **b** AP weightbearing pelvis radiograph demonstrates the same concept. The dot within the red circle represents the center of rotation for the femoral head, and the solid blue line reflects the anatomical axis of the femur. The femoral offset is the perpendicular distance between the center of the femoral head and the anatomical axis of the femur. Restoring femoral offset is critical to optimizing appropriate adductor muscle tension and maintaining hip stability following arthroplasty

Femoral offset restoration also plays a critical role in functional outcomes after arthroplasty. Measured on a weightbearing AP pelvis radiograph, the femoral offset is defined as the perpendicular distance from the femoral head center of rotation to a line along the femoral metaphysis long axis (Fig. 19) [73]. This measurement is sensitive to femoral rotation and femoral anteversion, with external rotation resulting in underestimation of the true lateralization value; thus, appropriately positioned radiographs with internal femoral rotation are critical for accurate measurements. The average femoral offset measured on radiography is approximately 38.7 ± 5.9 mm, though 3D CT analysis suggests that this may slightly underestimate the true values by approximately 3.5 ± 2.6 mm [74]. If the proximal femur has a dysmorphic appearance, the contralateral side can be used to estimate the appropriate ipsilateral femoral offset. However, this technique can result in measurement inaccuracy [75].

##### Vertical center of rotation

Measured on weightbearing AP pelvis radiographs, the vertical center of rotation is defined as the distance between the femoral head center of rotation and a horizontal line connecting the two ischial tuberosities (Fig. 18). The measured distance should be similar to the contralateral hip [72].

##### Horizontal center of rotation

Measured on weightbearing AP pelvis radiographs, the horizontal center of rotation is defined as the distance between the femoral head center and margin of the teardrop (Fig. 18). If the teardrop is not visible, the distance from femoral head center to Kohler’s line should be used [76]. The measured distance should be equal to the contralateral hip [72].

##### Radiographic lateral acetabular inclination

**Fig. 20:**
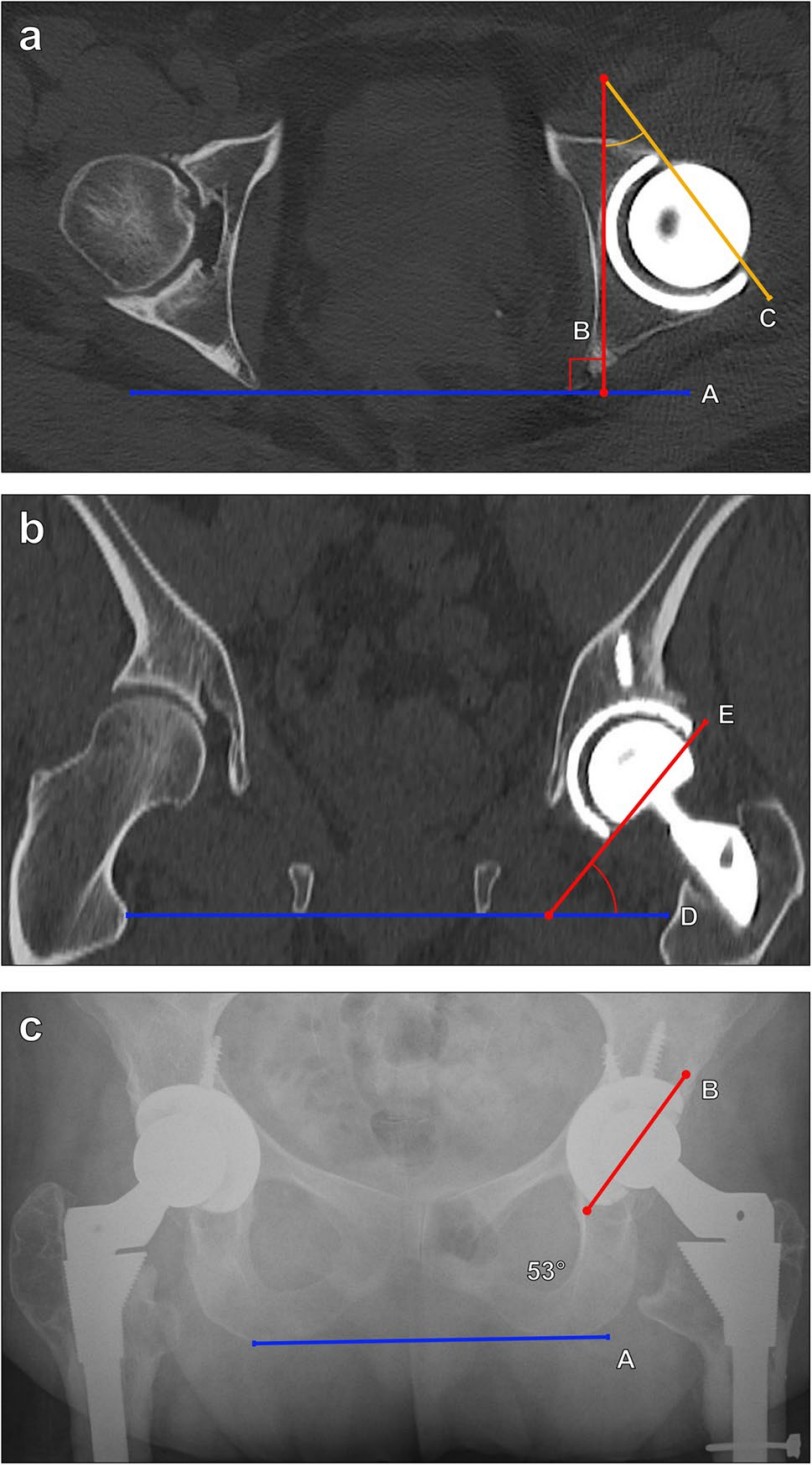
Acetabular cup anteversion is measured on axial CT images. **a** A line connecting the ischial tuberosities serves as the transverse long axis line (A, blue line). The version angle is formed between a line perpendicular to the transverse long axis (B, red line) and a line connecting the anterior and posterior rims of the acetabular cup (C, yellow line). **b** Acetabular cup inclination measured on coronal CT images or AP pelvis radiographs. The angle is formed between a line connecting the ischial tuberosities serving as the horizontal long axis of the pelvis (D, blue line) and a line along the rim of the acetabular cup (E, red line). Ideal acetabular cup positioning is about 15º–20° of anteversion and 40° of inclination. **c** On an AP pelvic radiograph of the pelvis coned to the hips in a 55-year-old patient with left hip pain, the acetabular cup inclination measures 53°, which is slightly elevated, which may predispose the patient to acetabular dislocation. One month later, the patient returned to the Emergency Department with a superior hip dislocation after minimal trauma

Acetabular inclination evaluates the angle between the face of the acetabular cup and the transverse axis of the body. Measured on AP pelvis or hip radiographs, this is defined as the angle formed between a line along the acetabular cup rim and a horizontal line connecting the ischial tuberosities (or teardrops) (Fig. 18) [77]. The normal value should be 40° ± 10° [78]. Lower values can result in limited abduction, and higher values imply increased risk of hip dislocation. An example of an increased lateral acetabular inclination angle in a patient who subsequently presented with superior hip dislocation is seen in Fig. 20c.

##### Acetabular component positioning

There are many suggested techniques for evaluating acetabular cup anteversion on radiography with a range of reported normal values [79]; however, this measurement is very sensitive to even slight changes in pelvic tilt, leg rotation, and x-ray beam angle, and if accurate measurement is required, it should be performed on CT.

Acetabular cup anteversion is measured on axial CT images through the center of the acetabular cup component (Fig. 20a). First, the transverse long axis of the pelvis is defined by a line connecting the ischial tuberosities. The acetabular angle is formed by a line perpendicular to the transverse long axis and a line connecting the anterior and posterior rims of the acetabular cup. Optimal cup positioning was described by Lewinnek et al. in 1978 as the “Lewinnek safe zone” which is defined as acetabular cup anteversion of 15° ± 10° [80]. Excessive acetabular cup anteversion may increase the risk of anterior hip dislocation, while reduced anteversion or even retroversion can lead to anterior impingement and decreased range of motion.

Acetabular cup inclination is measured on coronal CT images of the pelvis or AP pelvis radiographs (Fig. 20b). The angle is formed between a line connecting the ischial tuberosities serving as the horizontal axis of the pelvis and a line along the rim of the acetabular cup. As described by Lewinnek, optimal abduction measures 40° ± 10° [80]. Straying from the “Lewinnek safe zone” range increases the risk of hip dislocation.

##### Femoral stem positioning

The femoral stem should be in neutral position within the shaft, with slight anteversion of the neck. On an AP radiograph, a line along the long axis of the stem should align with the anatomic axis of the femur, and the prosthetic tip should be in the center of the medullary space [81]. Varus positioning of the femoral component has been associated with loosening and prosthetic failure [82].

##### Femoral stem subsidence

**Fig. 21:**
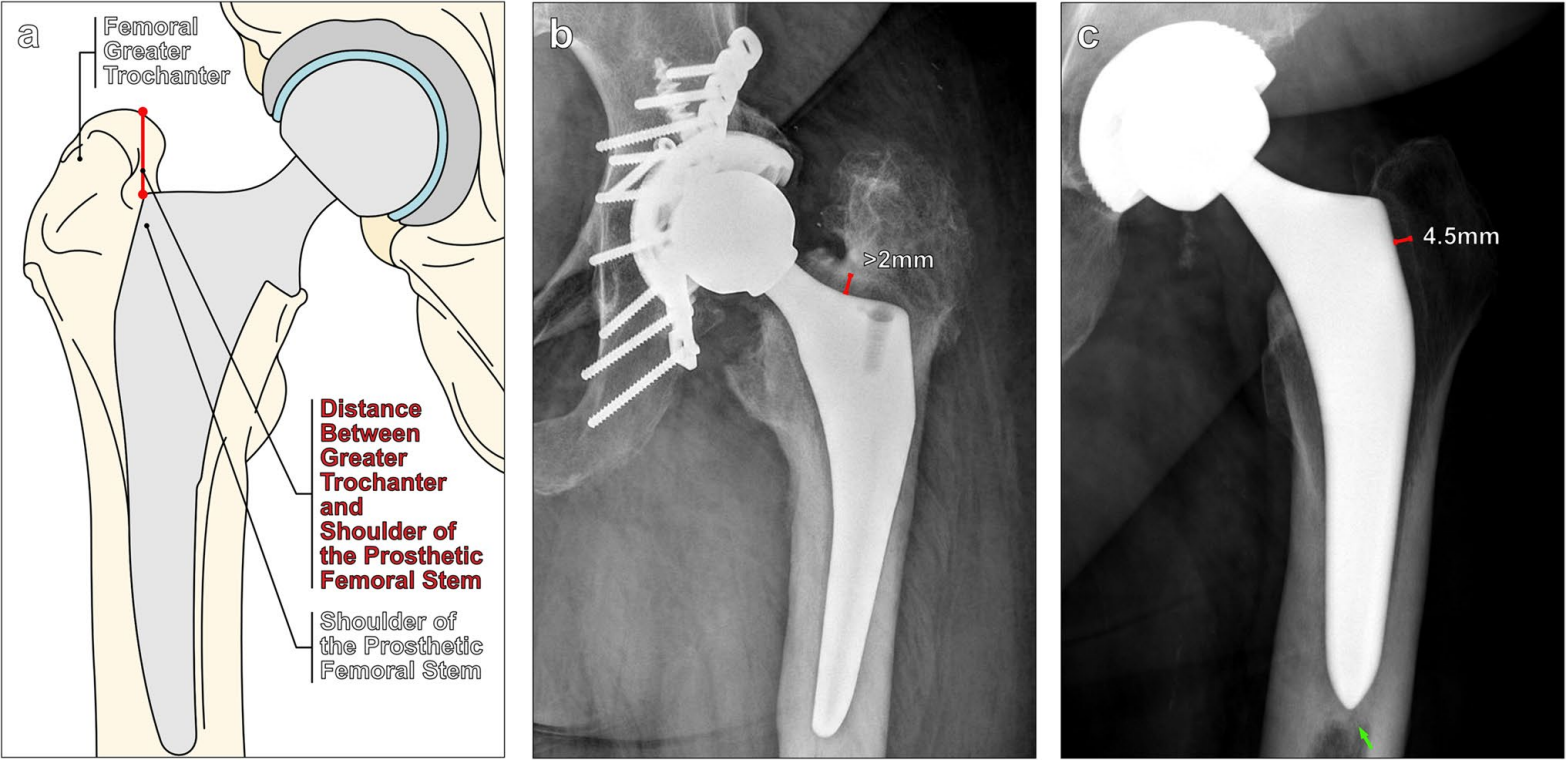
Subsidence and loosening. **a** Frontal diagram of the hip with total hip arthroplasty. The reference points to establish baseline positioning are commonly the superior tip of the femoral greater trochanter and the shoulder of the femoral prosthesis, which can be compared on serial examinations. **b** Measurement of subsidence on AP radiograph of the hip demonstrating lucency along the superolateral aspect of the femoral prosthesis measuring > 2 mm. Subsidence is determined by the inferior migration of the femoral prosthesis relative to fixed bony landmarks; periprosthetic lucency > 2 mm is considered significant and may indicate implant failure. **c** AP radiograph of the left hip in a 75-year-old male patient with total hip arthroplasty shows excessive periprosthetic lucency between the femoral component and the greater tuberosity, suggesting loosening. At the tip of the prosthesis, there is a transverse sclerotic band, called the *pedestal sign* (arrow), which can occasionally be seen with loosening

Subsidence is assessed serially on AP hip radiographs by measuring the vertical distance between fixed points on the proximal femur and the femoral prosthesis. The superior tip of the femoral greater trochanter and the shoulder of the prosthesis are common points of reference. If the shoulder of the femoral prosthesis is not visible, the distal tip of the prosthesis can be used. During the first 2 years following total hip arthroplasty, 1–2 mm of subsidence is normal for some types of prostheses and is often seen superolaterally. While subsidence > 3 mm may be significant and should be reported, subsidence > 5 mm may require intervention. If it is > 10 mm, it is considered extreme. Additionally, any progressive movement after 2 years is considered abnormal (Fig. 21) [83].

##### Loosening

A thin radiolucent band with an outer thin sclerotic line running parallel to the femoral stem is a common finding and is a result of a fibrous membrane forming between the cement and adjacent bone, or fibrous ingrowth into uncemented components. This membrane can measure up to 2 mm and becomes stable after 2 years following surgery. Any increase in thickness beyond 2 mm, or progression after 2 years is considered abnormal (Fig. 21). Additionally, a thin well-demarcated radiolucent band along the distal tip of the femoral stem is related to micromotion and is considered normal if ≤ 2 mm and stable [83].

##### Stress shielding

Wolff’s law is a fundamental property of bone in which bone placed under greater mechanical load will adapt to become stronger and thicker, while bone that is placed under decreased stress will become weaker. Following hip arthroplasty, there is alteration in the normal biomechanics of the hip with decrease in load-bearing of the proximal femur as weight is transferred to the prosthesis. This can result in the phenomenon of stress shielding, in which osteoporosis develops within the periprosthetic bone. The risk for stress shielding is greater with uncemented arthroplasties. Radiographically, stress shielding results in lucency with loss of periprosthetic bone mass in the proximal femur. While some degree of stress shielding is expected, more advanced cases predispose to periprosthetic fracture and prosthetic loosening [84].

## Conclusion

Knowledge of measurements on diagnostic imaging can guide diagnosis, treatment options, and surgical management of many hip disorders. There are numerous validated measurements which can be used in many clinical scenarios, particularly when evaluating lower extremity alignment, hip instability and biomechanical causes of pain, and postsurgical hip arthroplasty evaluation. These measurements require rigorous adherence to standardized patient positioning and imaging technique to obtain accurate and reproducible values that can be used across institutions. The decision of which measurements to obtain depends on the clinical scenario, and discussion with orthopedic surgeons may be helpful to determine which measurements they find most useful for their practice. This review article describes many of the most commonly utilized measurements to quantitatively assess the hip on imaging.

## Data Availability

Not applicable.
